# Vascular Stiffness in Aging and Disease

**DOI:** 10.3389/fphys.2021.762437

**Published:** 2021-12-07

**Authors:** Stephen F. Vatner, Jie Zhang, Christina Vyzas, Kalee Mishra, Robert M. Graham, Dorothy E. Vatner

**Affiliations:** ^1^Department of Cell Biology and Molecular Medicine, Rutgers University – New Jersey Medical School, Newark, NJ, United States; ^2^Victor Chang Cardiac Research Institute, University of New South Wales, Darlinghurst, NSW, Australia

**Keywords:** aortic stiffness, aging, cardiovascular diseases, human, non-human primate

## Abstract

The goal of this review is to provide further understanding of increased vascular stiffness with aging, and how it contributes to the adverse effects of major human diseases. Differences in stiffness down the aortic tree are discussed, a topic requiring further research, because most prior work only examined one location in the aorta. It is also important to understand the divergent effects of increased aortic stiffness between males and females, principally due to the protective role of female sex hormones prior to menopause. Another goal is to review human and non-human primate data and contrast them with data in rodents. This is particularly important for understanding sex differences in vascular stiffness with aging as well as the changes in vascular stiffness before and after menopause in females, as this is controversial. This area of research necessitates studies in humans and non-human primates, since rodents do not go through menopause. The most important mechanism studied as a cause of age-related increases in vascular stiffness is an alteration in the vascular extracellular matrix resulting from an increase in collagen and decrease in elastin. However, there are other mechanisms mediating increased vascular stiffness, such as collagen and elastin disarray, calcium deposition, endothelial dysfunction, and the number of vascular smooth muscle cells (VSMCs). Populations with increased longevity, who live in areas called “Blue Zones,” are also discussed as they provide additional insights into mechanisms that protect against age-related increases in vascular stiffness. Such increases in vascular stiffness are important in mediating the adverse effects of major cardiovascular diseases, including atherosclerosis, hypertension and diabetes, but require further research into their mechanisms and treatment.

## Introduction

The goal of this article is to review what is known about changes in vascular stiffness with aging and disease. It is widely accepted that aortic stiffness increases with advancing age. However, most existing research employs measures of aortic stiffness at a single aortic location as an estimate of overall aortic stiffness. This makes it a challenge to understand age-related stiffness along the length of the aortic tree, from the aortic root to its bifurcation into the iliac arteries, and regional vessels. Another goal is to review human and non-human primate data, which is particularly important for understanding sex differences in vascular stiffness with aging and the changes in vascular stiffness before and after menopause in females. Several mechanisms that mediate the increases in vascular stiffness will be reviewed. The most well-studied mechanism involves the extracellular matrix, with increases in vascular collagen and decreases in vascular elastin. There are other mechanisms, less well studied, that also contribute to the increased vascular stiffness, e.g., collagen and elastin disarray and increased vascular smooth muscle cell stiffness and numbers. Further insight can also be gained from populations with an extended lifespan, living in areas called “Blue Zones,” where a healthy diet and exercise ameliorate the increases in vascular stiffness observed with age.

## Aorta

### Anatomy

The aorta is divided into sections by location; the ascending aorta, aortic arch, and the descending aorta. The descending aorta can be divided into thoracic and abdominal sections. Branches of interest include the left and right coronary arteries, which branch from the aortic root, and the brachiocephalic, left carotid, and left subclavian, which branch from the aortic arch. As the aorta descends there are numerous branches which supply the surrounding muscles and organs including intercostal, celiac, hepatic, gastric, splenic, renal, mesenteric, and gonadal arteries. The abdominal aorta bifurcates into the iliac arteries which extend inferiorly, turning into the femoral arteries, which supply blood flow to each leg.

### Morphometry

The morphometric properties of the aorta differ along its length. The aorta tapers, with the average systolic diameter decreasing from the proximal to the distal aortic tree (Hickson et al., 2010). In healthy humans, helical computed tomography showed that maximum aortic diameter is in the ascending aorta, distal to the aortic valve sinus and proximal to the innominate artery (Hager et al., 2002). The aortic diameter then decreases progressively along the thoracic aorta and continues to decrease from the infrarenal abdominal aorta to the lower abdominal aorta (Hager et al., 2002; Rogers et al., 2013). Overall thickness of the aortic wall also decreases down the thoracic aorta, but then remains constant in the abdominal aorta (Sokolis, 2007). In the pig, the tunica media decreases in thickness distally along the thoracic and abdominal aorta, while the tunica adventitia thickness is negligible in the thoracic aorta, but increases down the length of the abdominal aorta (Sokolis, 2007). Aging results in morphometric changes to the diameter, length, and thickness of the aorta. Overall, the diameter and length increase progressively with age (Komutrattananont et al., 2019), with the greatest change in diameter occurring at the level of the ascending aorta (+0.96 mm/decade) (Hickson et al., 2010). The tunica intima and media of the aortic wall thicken with age (Komutrattananont et al., 2019).

## Age-Related Changes in Aortic Stiffness

One measure of aortic stiffness, carotid-femoral pulse wave velocity (PWV), is an estimate of the pulse transit-time between the carotid and femoral arteries (O’Rourke et al., 2002; Pannier et al., 2002; Laurent and Boutouyrie, 2020). This is an approximation that averages the many branches of the aortic tree and does not consider the influence of regional differences in stiffness and diameter (Millasseau et al., 2005). With aging, large elastic arteries, such as the aorta, show increases in arterial stiffness, which correlate with histological and biochemical changes within the arterial wall. Several studies have examined both thoracic and abdominal aortic stiffness with aging, *in vivo* (Farrar et al., 1984; Rogers et al., 2001; Nelson et al., 2009; Hickson et al., 2010; Taviani et al., 2011; Westenberg et al., 2011; Devos et al., 2015). In humans, where PWV was measured by cine phase contrast magnetic resonance imaging (PCMRI) in four segments of the aorta, it was found that the greatest age-related increase in aortic stiffness occurred in the abdominal aorta (+0.9 m/s per decade) followed by the thoracic-descending region (+0.7 m/s), the mid-descending region (+0.6 m/s), and aortic arch (+0.4 m/s) (Hickson et al., 2010). Another study, focusing on the ascending, descending, and infrarenal aorta showed increases in stiffness down the aortic tree in humans aged 40, 60, and 75 years (Cuomo et al., 2017). Variation in stiffness down the aortic tree has also been addressed via computational modeling of the human aortic tree using several metrics for stiffness and geometric and hemodynamic data from the literature. *In silico* examination of the effect of aging showed that pulse pressure and stiffness increase down the aortic tree and are most marked with advanced age. PWV may deviate from this pattern when it comes to the most distal sections of the aorta, as these are likely influenced by arterial tapering and branching (Cuomo et al., 2017). Isolated *in vitro* studies have also found that abdominal aortic stiffness is increased more with aging (Haskett et al., 2010).

Although many studies have reported that stiffness increases down the aortic tree, there still is some controversy. Some *in vivo* studies reported that increases in thoracic aortic stiffness with aging were greater than, or similar to those observed in the abdominal aorta (Farrar et al., 1984; Rogers et al., 2001; Nelson et al., 2009; Hickson et al., 2010; Taviani et al., 2011; Westenberg et al., 2011; Devos et al., 2015). Using PCMRI in a single para-sagittal plane to measure PWV in different regions, one study in humans found that participants below 55 years of age had similar PWVs at different aortic locations, but those older than 55 experienced the reverse of what is generally thought, i.e., stiffness decreased down the aortic tree (Rogers et al., 2001). In this study the investigators also suggested that the most significant mechanisms for increasing aortic stiffness with age are fragmentation of elastin, which would primarily affect the proximal aorta due to its higher elastin content, and diminished nitric oxide activity (Rogers et al., 2001). However, it’s possible that the changes noted are not statistically significant, since the population studied had a low probability of having atherosclerosis and PWV variability increased markedly with age. Another study in humans found no significant difference in aortic PWV in pre- and post-menopausal women, but significantly lower brachial and femoral PWV values in pre-menopausal women (London et al., 1995).

**FIGURE 1:**
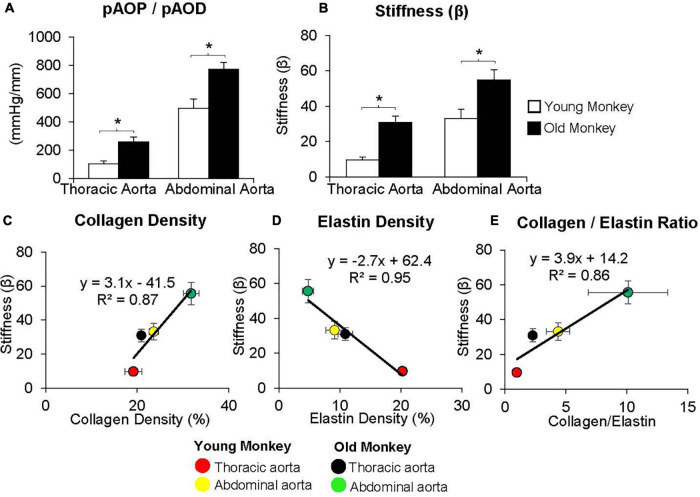
**(A)** Measurements of the ratio of pulse aortic pressure (pAoP) to pulse aortic diameter (pAoD) are compared in young and old male monkeys both in the thoracic aorta and the abdominal aorta. The pAOP/pAOD ratio was significantly increased in old male monkeys, both in the thoracic and abdominal aorta. **(B)** Aortic stiffness was significantly increased in both the thoracic and abdominal aorta in old (26 ± 1 years) compared to young (9 ± 1 years) male monkeys. Correlation between aortic stiffness and collagen density **(C)**, elastin density **(D)**, and the collagen/elastin ratio **(E)** show linear relationships with aortic stiffness. Aortic stiffness was higher in the abdominal aorta than in the thoracic aorta in both young and old male monkeys and was higher in old vs. young male monkeys in both the thoracic and abdominal aorta. *p < 0.05 vs. corresponding young animals (reprint from Zhang et al., 2016).

**FIGURE 2:**
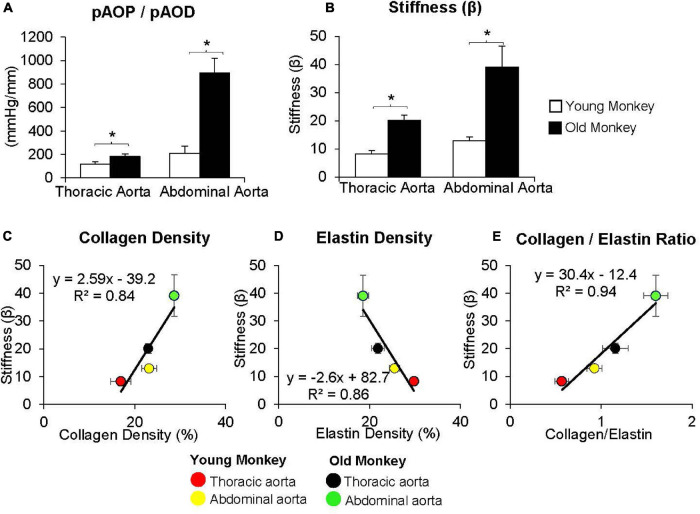
**(A)** Measurements of the ratio of pulse aortic pressure (pAoP) to pulse aortic diameter (pAoD) are compared in young and old female monkeys both in the thoracic aorta and the abdominal aorta. The pAOP/pAOD ratio was significantly increased in old female monkeys, both in the thoracic and abdominal aorta. **(B)** Aortic stiffness was significantly increased in both the thoracic and abdominal aorta in old (24 ± 0.7 years) compared to younger (7 ± 0.7 years) monkeys. Correlation between aortic stiffness and collagen density **(C)**, elastin density **(D)**, and the collagen/elastin ratio **(E)** show linear relationships with aortic stiffness. Aortic stiffness was higher in the abdominal aorta than in the thoracic aorta in both old and young monkeys and was higher in old vs. young monkeys in both the thoracic and abdominal aorta. *p < 0.05 vs. corresponding young animals (reprint from Babici et al., 2020).

**FIGURE 3:**
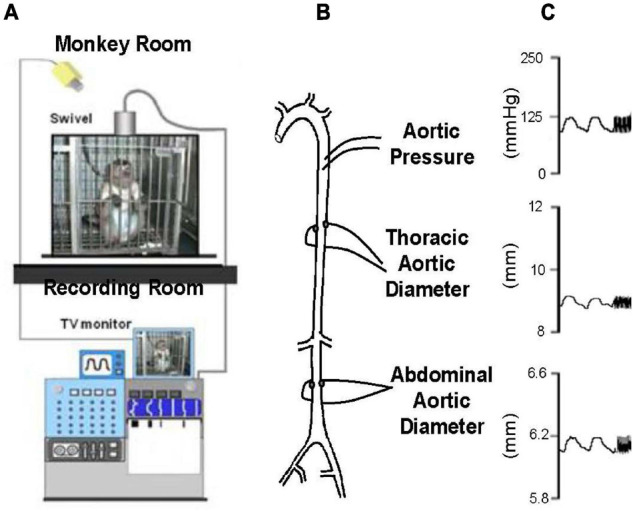
**(A)** Chronically instrumented, conscious monkeys were connected to a tether to record data, but otherwise unrestrained in their cage during recording. **(B)** The animals were instrumented with thoracic aortic catheters for measurement of aortic pressure and ultrasonic dimension crystals, on opposing surfaces of the thoracic and abdominal aorta, for measurement of aortic diameters. **(C)** Examples of phasic measurements of aortic pressure and diameters in a young adult monkey (reprint from Zhang et al., 2016).

By comparison with these studies in humans, we found significantly greater increases in stiffness in the abdominal aorta compared to the thoracic aorta in studies of non-human primates (Zhang et al., 2016; Babici et al., 2020; Figures 1, 2). Recording of aortic dimensions using implanted ultrasonic crystals in monkeys (Figure 3) showed that abdominal aortic stiffness was greater than thoracic aortic stiffness in both young and old monkeys (Figures 1, 2; Zhang et al., 2016). These measurements of arterial stiffness using direct and continuous measurements of arterial pressure and diameter are more precise than measurements of stiffness using PWV, since they permit assessment of stiffness at distinct locations in the aorta. Examination of old premenopausal female monkeys also showed that the aortic stiffness index (β) was significantly higher in the abdominal vs. the thoracic aorta, both in older (20 ± 1.8) and younger (8 ± 1.1) monkeys (Babici et al., 2020). In addition, histological correlates of vessel stiffness were greatest in the iliac artery, suggesting iliac artery stiffness was even greater than abdominal aortic stiffness (Babici et al., 2020). Our *in vivo* studies of monkeys clearly indicate that age-related increases in aortic stiffness are greater in the abdominal compared to the thoracic aorta (Figures 1, 2). Our previous studies also found significantly greater aortic stiffness in the abdominal compared to the thoracic aorta, in young monkeys (Zhang et al., 2016; Babici et al., 2020). Similarly, in normal rabbits PWV increased more with age in the abdominal than the thoracic aorta (Katsuda et al., 2014).

As noted above there are several reasons why studies in non-human primates are ideal for understanding vascular stiffness. Although it would be best to conduct these studies in humans there are ethical limitations to those studies and it is challenging to study changes in vascular stiffness in the absence of other disease states that normally evolve in older patients. The non-human primate is closest to humans on the evolutionary tree and therefore has the closest changes in genomics to humans among animal models, which occur with age. Moreover, sex-specific changes with aging, particularly the similarity between menopause in humans and non-human primates, is another important feature. On the other hand, there are features that make it considerably more difficult to study non-human primates than other animal models. First of all cost: there is a difference of thousands of dollars in purchasing non-human primates compared to other laboratory animals. Secondly, their supply is limited. Thirdly, care for these animals is more complex and most vivariums do not have the appropriate veterinary staff and facilities to house primates. Finally, there is increasing criticism for the use on non-human primates on an ethical basis. Whereas, there are some groups that do not condone any animal research, there are others that specifically oppose primate research.

**FIGURE 4:**
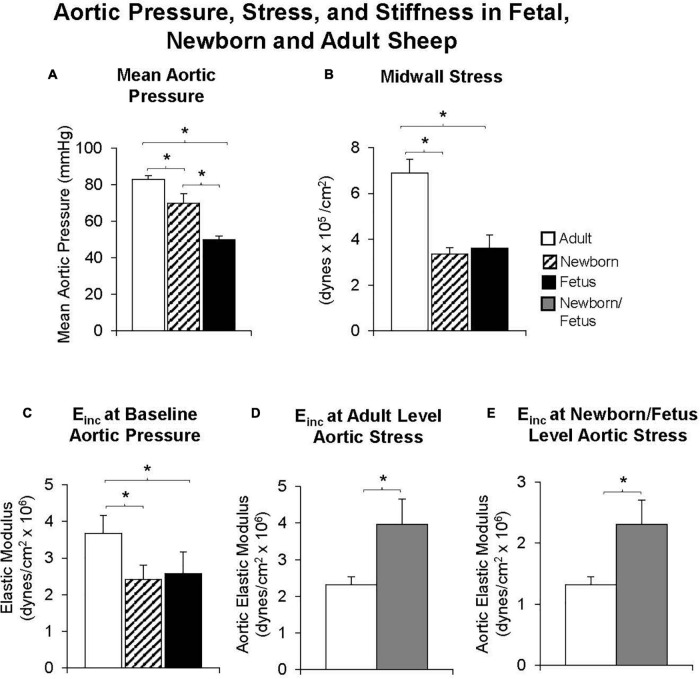
Both **(A)** mean aortic pressure and **(B)** midwall stress were lower in fetal or newborn lambs than in adult sheep. **(C)** The elastic modulus was lower in fetal and newborn lambs, compared with adult sheep at baseline levels of aortic pressure, but surprisingly, the reverse was observed when the elastic modulus was compared at equal levels of aortic stress in fetal and newborn lambs and adult sheep **(D)** or when compared at the fetal/newborn levels of aortic stress **(E)**. Moreover, values were similar in fetal and newborn lambs suggesting that increases in aortic stiffness occur well after birth. *p < 0.05 (replot from Pagani et al., 1979).

Most interest in age-related vascular stiffness has focused on the changes observed between midlife and older age in adults. However, it would also be of interest to know if changes in vascular stiffness occur between birth and midlife. To this end, we examined fetal, newborn and adult sheep, chronically instrumented for measurements of aortic diameter and pressure. At baseline levels of arterial pressure the elastic modulus of the aorta of young adult sheep was higher than that of the newborn lamb or the fetus, associated with a higher stress level (Pagani et al., 1979). However, when data were evaluated at common levels of stress, the aorta of the adult had a lower elastic modulus, than either the newborn or fetal animals (Figure 4). Furthermore, in the adult, a marked shift in the pressure-diameter and stress-radius relationships were observed in response to alpha-adrenergic mediated vasoconstriction. In contrast, no shift was observed in the newborn or fetal lambs (Pagani et al., 1979). The mechanism could either be at the level of either alpha adrenergic receptor signaling development or the inability of the aortic smooth muscle to constrict.

## Peripheral and Regional Vessels

In comparison to the aorta, peripheral arteries are less elastic, more muscular, and inherently stiffer (Yu and Mceniery, 2020). In humans, the femoral artery has a more rigid wall, a greater diastolic diameter, and a twofold lower distensibility coefficient than the carotid artery (Benetos et al., 1993). The diastolic diameter of the carotid artery increases with age, while in the femoral artery, arterial diameter only increases slightly with age (Benetos et al., 1993). Carotid artery distensibility decreases linearly with aging, and cross-sectional compliance also decreases (Benetos et al., 1993). Although stiffness of peripheral arteries prior to the age of 50 is higher than that of central arteries, the increases in stiffness with aging is less in peripheral arteries than in central arteries (Mitchell et al., 2004). A study of static mechanical properties using an ultrasonic phase locked echo tracking system showed that the common carotid, femoral, and brachial arteries all increase in diameter with age (Kawasaki et al., 1987). Stiffness increases in all arteries as well; however, this was only significant in the common carotid and changes in stiffness of the brachial and femoral arteries varies greatly among individuals (Kawasaki et al., 1987). As with the aging aorta, it is believed that collagen content in the pulmonary and carotid arteries increases, while elastin content and the number of vascular smooth muscle cells (VSMCs) decrease with age (Greenwald, 2007). However, aging likely impacts large conduit arteries, such as the aorta and carotid, differently than small resistance vessels, which have fewer layers of VSMC and less matrix (Trache et al., 2020).

In non-human primates, as noted above, stiffness increases from the thoracic to the abdominal aorta and even distally to the iliac artery in both male and female monkeys (Zhang et al., 2016; Babici et al., 2020). However, most other studies have not measured stiffness directly but have examined the histological properties at different vessel levels. For example, the canine femoral artery has a higher content of collagen and lower content of elastin when compared to the ascending aorta, which would correlate to increases in stiffness physiologically (Fischer and Llaurado, 1966). However, stiffness was not directly measured in this study at any of the sites analyzed (Fischer and Llaurado, 1966).

## Mechanisms of Age-Related Increases in Vascular Stiffness

**FIGURE 5:**
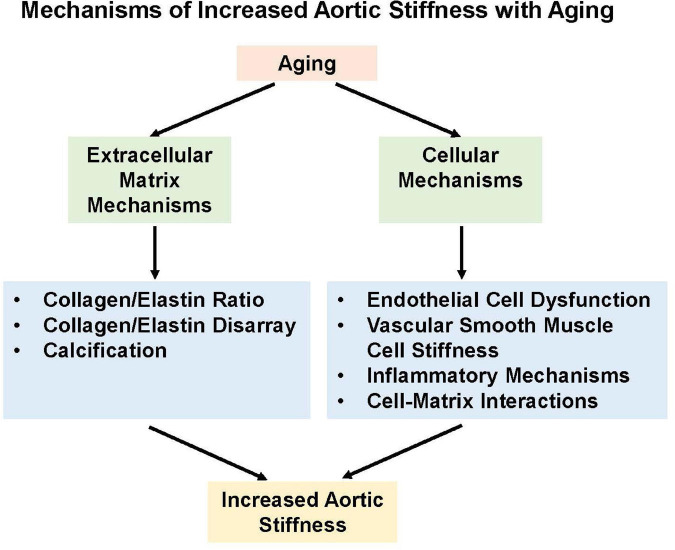
Increased arterial stiffness develops from both extracellular and cellular mechanisms.

The mechanisms of increased stiffness in aging are both extracellular and cellular (Figure 5). The three main aortic wall components, elastin, collagen, and smooth muscle cells, vary along the length of the aortic tree. With aging, these components of the aortic wall are altered. The number of elastic fibers and smooth muscle cells in the tunica media decrease, while collagen fibers increase with advancing age (Maurel et al., 1987). The number of smooth muscle cells in the tunica media decreases with age and vascular smooth muscle cell migration from the tunica media thickens the intima (Collins et al., 2014).

### Extracellular Matrix Remodeling

The most important mechanism studied as a cause of age-related increases in vascular stiffness is alteration in the extracellular matrix (ECM), resulting from an increase in collagen and decrease in elastin. The ECM is composed of a complex network of different matrix proteins, metalloproteases, and glycosaminoglycans, which are also responsible for the structural integrity of the vasculature, and therefore contribute to its stiffness (Ma et al., 2020). Collagen is a very stiff protein with the function of limiting vessel elasticity and distension (Briones et al., 2010), and is therefore fundamental to defining the stiffness of the arterial wall. Collagen deposition throughout the vasculature increases with age, which alters the normal ECM network (Kohn et al., 2015). This has been shown to occur in the intima, media, and adventitia of the vessel wall leading to substantial changes in its morphology and function (Greenberg, 1986; Fleenor et al., 2010, 2012). In addition to increased collagen deposition, there is also increased non-enzymatic glycation. This is also responsible for age-related increases in arterial stiffness (Schleicher et al., 1997), as it induces collagen cross-linking, which increases stiffness (Reddy, 2004).

Unlike collagen, elastin, the other major ECM protein, provides flexibility and extensibility of the vessel wall (Wagenseil and Mecham, 2012). Elastin fibers are mainly found in the medial layer of large elastic arteries and are oriented around smooth muscle cells and collagen. Elastin content decreases, while collagen content increases from the proximal to distal aorta (Fischer and Llaurado, 1966; Sokolis, 2007). The elastin/collagen ratio is highest in the thoracic aorta and decreases distally (Hager et al., 2002), whereas the reverse is found in the collagen/elastin ratio (Figures 1, 2). Smooth muscle cell content remains similar throughout the length of the aorta, but increases with aging and is another mechanism for increased aortic stiffness. Degradation of elastin fibers with aging is mediated by the increases of proteolytic enzymes, e.g., matrix metalloproteases (MMP), which degrade elastin fibers, resulting in an increase in collagen/elastin ratio, which in turn increase vessel wall stiffness (Wolinsky, 1970). Nevertheless, the extent to which increases in collagen and decreases in elastin contribute to increased vascular stiffness with aging remains controversial.

**FIGURE 6:**
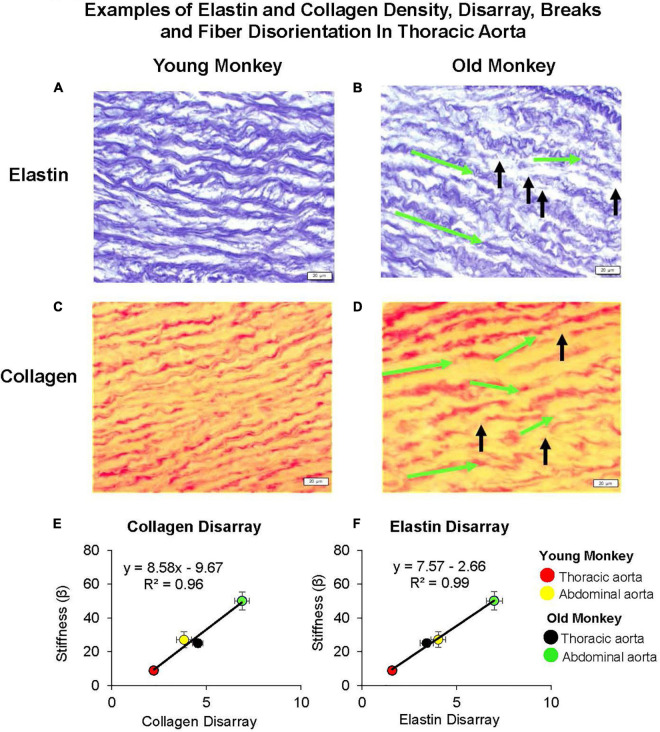
Examples of elastin and collagen fiber disarray, breaks, and fiber disorientation in the thoracic aorta of young **(A,C)** as compared to those of old premenopausal females **(B,D)**. In old premenopausal monkeys, elastin and collagen disarray, fiber breaks, and fiber disorientation were increased compared to the young females. The green arrows indicate the change of fiber angle from the starting point of a fiber toward the end point of a fiber, and the black arrows indicate the fiber breaks. A linear correlation between stiffness and extracellular matrix was found for both **(E)** collagen disarray and **(F)** elastin disarray. Disarray was greater in the abdominal vs. the thoracic aorta in both old and young female monkeys and both were greater in old female vs. young female monkeys (reprint from Babici et al., 2020).

Using old world monkeys, we previously found that collagen density in the thoracic aorta did not change with age, whereas that in the abdominal aorta increased. In contrast, we found that elastin in both the thoracic and abdominal aorta decreased with age (Zhang et al., 2016; Babici et al., 2020; Figures 1, 2, 6). Relatively few studies have examined changes in both thoracic and abdominal aortic stiffness with age, *in vivo* (Farrar et al., 1984; Rogers et al., 2001; Nelson et al., 2009; Hickson et al., 2010; Taviani et al., 2011; Westenberg et al., 2011; Devos et al., 2015). Some studies measured collagen and elastin with aging, but did not measure aortic stiffness, *in vivo*. Interestingly, the results of these studies are controversial, with some studies finding an increase (Faber and Oller-Hou, 1952; Chamiot-Clerc et al., 2001; Aronson, 2003; Nosaka et al., 2003; Astrand et al., 2011; Taviani et al., 2011; Wheeler et al., 2015) and others finding no change in collagen (Hosoda et al., 1984) or a decrease in collagen (Nosaka et al., 2003; Qiu et al., 2007a; Astrand et al., 2011; Taviani et al., 2011; Atanasova et al., 2012) or no change in elastin (Wheeler et al., 2015).

In contrast to the thoracic aorta, collagen content rose by 34% in the abdominal aorta with aging, which was a significantly greater increase than that observed in the thoracic aorta (Zhang et al., 2016). Elastin was decreased in the abdominal compared to the thoracic aorta in young animals, and decreased to even lower levels with aging (Zhang et al., 2016). Consistent with our finding that stiffness in the abdominal aorta of young monkeys equaled or was greater than that of the thoracic aorta in old monkeys, the collagen and elastin levels in the abdominal aortas of young monkeys equaled the values observed in the thoracic aorta for old monkeys (Zhang et al., 2016; Babici et al., 2020), emphasizing the importance for studying regional aortic stiffness changes with aging. However, basal levels of collagen and elastin are not the only mechanism accounting for greater stiffness observed in the abdominal compared to the thoracic aorta both in young and old monkeys. From our previous studies, we also observed marked disarray of both collagen and elastin; a finding that was more prominent with aging and in the abdominal vs. thoracic aorta. In fact, the marked disarray of elastin and collagen in the young abdominal aorta is likely responsible for the unexpected more severe stiffness than even in the old thoracic aorta (Zhang et al., 2016; Babici et al., 2020). The elastin and collagen disarray correlated better with stiffness than did elastin and collagen content (Figure 6). Another study also found elastic tissue in the abdominal aorta is most affected by aging (Maurel et al., 1987). Elastic fibers become damaged and thicken the tunica intima. Within the tunica media, elastic lamellae become damaged and elastic fibers become fragmented and disarrayed (Maurel et al., 1987). It is surprising that this marked architectural disarray we observed in the aging aorta with increased stiffness has not been noted extensively in the past, even though isolated observations have previously found disarray in aortae related to aneurysms (Hofmann Bowman et al., 2010; Pezzini et al., 2012; Lee et al., 2014), hypertension (Sans and Moragas, 1993), and aging (Fornieri et al., 1992).

Other important mechanisms mediating increased vascular stiffness, include increases in calcium deposition, endothelial dysfunction, and increases in the stiffness of vascular smooth muscle cells.

### Calcium Deposition

Calcification of the vessel wall occurs with normal aging, reducing the vessel wall’s distensibility (London et al., 2003). In humans there is a direct correlation between aortic calcification and arterial stiffness (Guo et al., 2017). Calcinosis of arterial walls with aging has been associated with increased cholesterol content in the elderly, suggesting a relationship between these processes (Kanabrocki et al., 1960). However, it is unknown which process occurs first, although some have speculated that calcinosis increases interaction with cholesterol molecules in the arterial wall (Hornebeck and Partridge, 1975). Another explanation for the increase in calcium deposition within the arterial wall is via an increase in inflammation and oxidative stress, both of which occur with normal aging. Increases in oxidative stress that occur with aging, mainly due to decreases in mitophagy and autophagy (Pescatore et al., 2019), stimulate vascular calcification by activating several signaling cascades (Byon et al., 2008). One of the best studied signaling pathways involves the upregulation of bone morphogenetic proteins due to increases in oxidative stress, which results in increased vascular calcification (Sorescu et al., 2004; Johnson et al., 2006).

The relationship between calcification and aortic stiffness differs depending on the location of the calcification. Carotid artery stiffness was more strongly associated with thoracic aorta calcification than calcification of the coronary arteries (Blaha et al., 2009). This may be because calcification of the coronary arteries usually involves only the intimal layer, while in large arteries calcification involves both the intima and media (Blaha et al., 2009); with medial calcification being more strongly associated with aging, diabetes, and severe renal disease (Iribarren et al., 2000). In addition, structural features may be involved: carotid arteries, being more elastic, are more similar to the aorta than to coronary arteries, which are non-elastic, predominantly conduit vessels.

### Endothelial Dysfunction

The vascular endothelium is the innermost, monolayer of cells in blood vessels. When the endothelium is healthy, vascular tone is regulated by a balance of vasoconstriction and vasodilation; the latter controlled by nitric oxide (NO) release (Furchgott and Zawadzki, 1980). Reduced bioavailability of nitric oxide leads to endothelial dysfunction, resulting in impaired vasodilation, which increases arterial stiffness (Mceniery et al., 2006). Endothelium impairment and decreased NO bioavailability occur with normal aging, ultimately leading to a proinflammatory, vasoconstrictive state, resulting in increased vascular fibrosis and arterial stiffness (Santhanam et al., 2010; Santos-Parker et al., 2014). Furthermore, endothelial dysfunction leads to an increase in oxidative stress through an increase in the production of superoxide causing damage to the vessels leading to changes in hemodynamics (Donato et al., 2018). Recently, it has also been proposed that autophagy, the cellular housekeeping mechanism that maintains cellular homeostasis, decreases in the aging endothelium, further leading to increases in oxidative stress (Larocca et al., 2012). This was further confirmed with the use of a pro-autophagy treatment, which reduced arterial stiffness and oxidative stress in aged mice (Larocca et al., 2013).

Age-related endothelial dysfunction may affect the arterial network differently based on location and vessel type. Aging results in endothelial dysfunction in the aorta but not in the femoral artery (Barton et al., 1997). The anatomic variation in the influence of age on endothelial function may be related to increased pulse pressure and reduced eNOS mRNA expression in the aorta (Barton et al., 1997). In rats, acetylcholine-induced vasorelaxation is impaired in large conduit arteries (abdominal aorta and iliac arteries) but not in smaller conduit (femoral arteries) or resistance arteries (Luttrell et al., 2020).

### Vascular Smooth Muscle Cells

**FIGURE 7:**
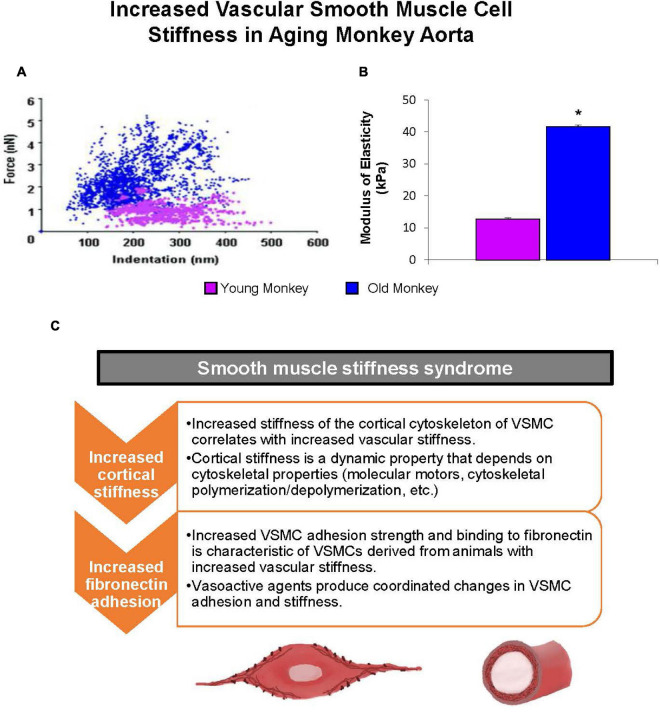
Mechanical properties of single VSMC measured by atomic force microscopy. **(A)** Distribution of force as a function of indentation in young (pink) (*n* = 40 cells) and old (blue) (*n* = 76 cells) monkeys. Increased cell stiffness is evident as higher force requirement for indentation. **(B)** VSMC stiffness increased fourfold more in old vs. young monkeys. *P < 0.05 vs. young monkeys. **(C)** Smooth muscle stiffness syndrome is characteristic of increased arterial stiffness and describes the aberrant increased stiffness and adhesion to fibronectin observed in vascular smooth muscle cells derived from stiff vessels (reprint from Sehgel et al., 2015b).

**FIGURE 8:**
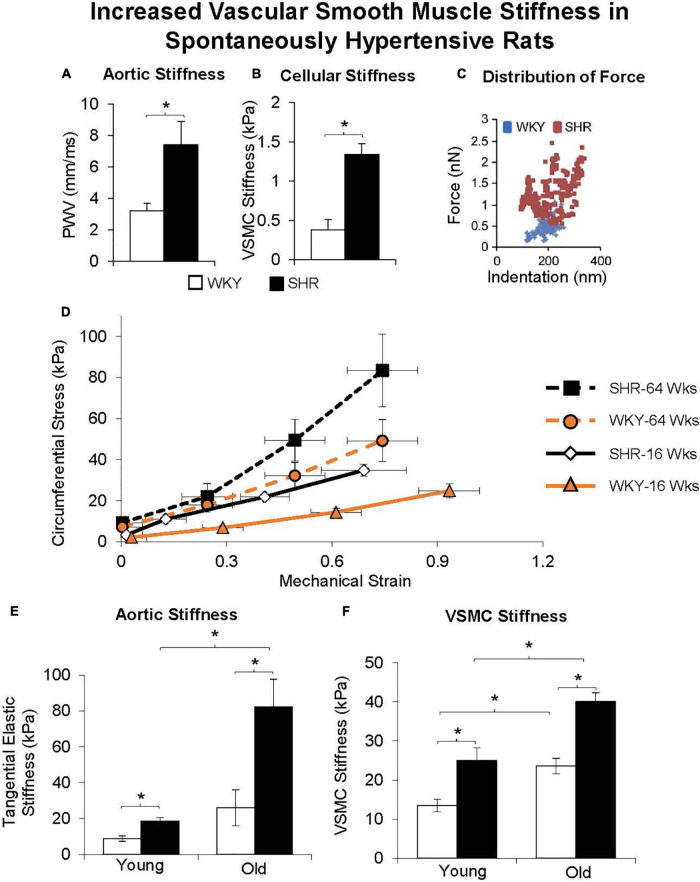
Aortic stiffness was increased in *in vivo* **(A)** and in vascular smooth muscle cells **(B)** in spontaneously hypertensive rats (SHRs) compared with Wistar-Kyoto (WKY) rats. **(C)** Distribution of force was also higher at all levels of indentation in old vs. young SHR. **(D)** Excised aortic rings from young (16 weeks old) and old (64 weeks old) WKY and SHR were mechanically stretched, and their stiffness was determined from the stress-strain relationship, as shown for representative rings. Stress was greater in old vs. young SHR and WKY rats at any level of strain. **(E)** Aortic stiffness was increased in the SHR more than WKY in young rats, and was further increased in old SHR. **(F)** VSMC stiffness was also increased more in SHR than WKY at both young and old ages, and with greater increases observed in old age. *p < 0.05 (reprint from Sehgel et al., 2013, 2015a).

Vascular smooth muscle cells have recently been discovered as important contributors to age-related increases in arterial stiffness (Trache et al., 2020). This mechanism, which we named “Vascular Smooth Muscle Cell Stiffness Syndrome” (Sehgel et al., 2015b), was elucidated in aged non-human primates that displayed an increase in VSMC stiffness in large arterial vessels (Qiu et al., 2010; Sehgel et al., 2015a; Figure 7). This increased VSMC stiffness is due to the direct relationship between VSMCs and endothelial cells. Endothelial cells regulate vascular tone mainly through the release of nitric oxide. This reduces active tone of VSMCs (Furchgott and Zawadzki, 1980; Van Bussel et al., 2015), which counteracts the increase in wall shear stress that occurs with both aging and high blood pressure (Boutouyrie et al., 1995; Van Bussel et al., 2015; Jaminon et al., 2019; Figure 8). However, aging also leads to a decrease in the number of cells within the vascular wall due to a decrease in cell proliferation with age (Greenwald, 2007; Chi et al., 2019). Multiple mechanisms mediate the decrease of VSMCs with age, but most notably inflammation and calcification, which increase VSMC apoptosis (Lacolley et al., 2012). In humans, the VSMCs lost with aging are replaced by collagen fibers in the media of the arterial wall, resulting in increased vascular stiffness (Schlatmann and Becker, 1977). Interestingly, when the stiffness of isolated VSMCs was measured with atomic force microscopy, it did not differ with age between the thoracic and abdominal aorta (Zhang et al., 2016). This suggests that VSMC stiffness is not the mechanism by which stiffness varies regionally within the aorta, unless it is related to differences in VSMC function as a result of altered endothelial cell function, inflammation or calcium mechanisms that occur variably along the aortic tree with aging.

## Extended and Abbreviated Lifespan Models

As emphasized in this review, increased aortic stiffness is mainly associated with aging. Interestingly, children with Hutchinson-Gilford progeria syndrome, a premature aging syndrome with a mean lifespan of 13 years, experience severe arterial stiffening from a young age. Patients aged 7 years have the aortic stiffness of a 60–69-year old without additional diseases (Gordon et al., 2005). This is thought to be related to abnormal elastin production (Sloop et al., 2015). These patients die prematurely, primarily of atherosclerotic disease and its complications. Since increased vascular stiffness, particularly in coronary arteries is linked to atherosclerosis, this provides further support for the concept that vascular stiffness is an important determinant of longevity.

Studying populations and animal models that experience longevity without cardiovascular decline and consequent increases in vascular stiffness could provide new insights into delaying vascular aging and promoting vascular health. Populations with extended lifespan live in areas known as Blue Zones (Vatner et al., 2020). They are geographically widespread, and include locations in the United States, Costa Rica, the Mediterranean, and East-Asia, Loma Linda, United States; the Nicoya peninsula in Costa Rica; Sardinia, Italy; Ikaria, Greece; Okinawa, Japan (Buettner and Skemp, 2016; Huang and Mark Jacquez, 2017). In these areas the number of centenarians, i.e., those reaching the age of 100, is 10 times greater than the average in the United States. The inhabitants share general features leading to healthful aging, e.g., frequent ambulation, healthy social relationships and psychological wellbeing, and diets that prevent weight gain, as opposed to Western diets, which are associated with increased vascular stiffness (Dupont et al., 2019). As noted above, protection against vascular stiffness plays a role in extended lifespan. Data supporting this can be found in a study in one of these Blue Zone populations, people living on the island of Ikaria, where aortic stiffness increases gradually with age, but then begins to decelerate at 50 years of age, with PWV being significantly lower than that in normal lifespan populations (Pietri et al., 2015). In the Ikaria population, habitual physical exercise, which is known to ameliorate the effects of vascular aging (Siasos et al., 2013), is associated with increased endothelial function, also known to protect against vascular stiffness (Anderson, 2006). It has also been shown that body fat-percentage is directly associated with arterial stiffness in long-lived populations, consistent with individuals with more lean muscle having more elastic arteries (Melo et al., 2021). Exercise also preserves muscle mass and a healthy diet can promote anti-inflammatory and anti-oxidative pathways (Redei and Mehta, 2015). Another interesting population are the Yanomami Indians of South America, whose diets are low in fat and salt, and high in fiber, plantains, cassavas (a root vegetable), and fruit. Vascular stiffness has not been studied in Yanomami Indians, but their blood pressure remains largely unchanged from age 1 to 60 years (Mueller et al., 2018).

Obesity, one of the most common worldwide problems, is associated with almost all cardiovascular diseases, including increased vascular stiffness (Dupont et al., 2019). Conversely, caloric restriction has also been reported to increase lifespan, both in humans and animal models, and protects against obesity, diabetes, hypertension, cancer, and cardiovascular disease (Trepanowski et al., 2011; Yan et al., 2012, 2013; Ravussin et al., 2015; Redman et al., 2018; Vatner et al., 2020). Caloric restriction also protects against arterial stiffness. In rats, this is evidenced by increased aortic distensibility and decreased PWV (Ahmet et al., 2011). Less collagen builds up, more elastin remains, and vascular smooth muscle is preserved in the aorta (Fornieri et al., 1999). A major mechanism by which caloric restriction is protective and prolongs longevity is through increased eNOS levels, which increase nitric oxide bioavailability, and protect against oxidative stress (Wilkinson et al., 2002).

However, vascular stiffness has not been evaluated in most studies of animal models with an extended lifespan. Whales, for example, are some of the longest living mammals but not much is known about the aging of their vasculature. They have a vastly different structure to their aortic tree with an anatomy representing arterial adaptation by diving mammals (Shadwick and Gosline, 1994). Of interest, Japanese women, who are lifelong pearl divers, demonstrate significantly lower arterial stiffness in proximal and elastic arteries and lower carotid artery impedance modulus compared with non-diving residents in the same village (Sugawara et al., 2018). Vascular stiffness, however, has not been studied in other long-lived animal models, such as, bats (Podlutsky et al., 2005), and tortoises (living over 100 years) (Quesada et al., 2019).

An exception to this is the naked mole-rat, which is the longest-lived rodent known (Dammann and Burda, 2006; Grimes et al., 2014). This rodent does not display the age-related pathology seen in other mammalian species, including shorter living rodents. In naked mole-rats, systolic, mean, and pulse pressure as well as PWV remain unchanged with age (Grimes et al., 2014). Additionally, they maintain normal cardiovascular structure and function at 24 years of age, which is 8 times the lifespan of normal rats, an age physiologically equivalent to a 92-year-old human (Grimes et al., 2014). Studies suggest that their youthful vasculature may be attributed to sustained nitric oxide availability and protection against oxidative stress (Csiszar et al., 2007). Further studies of vascular stiffness in animal models with extended or abbreviated lifespan are warranted as they might provide novel therapeutic targets for the prevention of age-related increases in vascular stiffness.

## Sex Differences

Aging-related vascular stiffening is sexually dimorphic, a topic that has not been studied extensively. Although arterial stiffness increases from young adulthood to older ages in both men and women, the increases in stiffness in women before menopause are less than those in age-matched men (Ogola et al., 2018). This pattern reverses after menopause (Nethononda et al., 2015; Babici et al., 2020). This may partly explain why women tend to live longer than men but are in worse health at older ages when compared to men (Hagg and Jylhava, 2021). In humans, postmenopausal women have a higher carotid-femoral PWV than premenopausal women; a step-up in PWV that is not observed in age-adjusted men (Staessen et al., 2001). This finding suggests that investigations into sex-specific gene regulation and sex hormones, may provide new insights into the mechanisms mediating these patterns.

Much of the previous experimental work on vascular stiffness has been performed in rodent models, which have a short lifespan and do not experience menopause. Because of this, the most relevant studies are those in non-human primates and humans. Non-human primates have a longer lifespan (>30 years), and undergo menopause like humans but are exempt from diseases, such as atherosclerosis, hypertension and diabetes, which are confounding factors when studying age-related changes in vascular stiffness in humans (Qiu et al., 2007a).

The studies considered here, on sex differences in arterial stiffness in humans, will focus predominantly on primary measures of arterial stiffness, namely, PWV, pulse pressure, and aortic distensibility; PWV and pulse pressure increasing and aortic distensibility decreasing as age and stiffness increases. In a study involving 777 people aged 21–85 years, aortic distensibility and aortic PWV were assessed using cardiovascular MRI, with age-related differences examined in successive deciles in each sex (Nethononda et al., 2015). In the first age group (20–29 years), aortic distensibility was significantly higher in women than men. This pattern was reversed in older aged groups, the sex differences being most marked in those aged around 60 years. In both sex groups, aortic distensibility decreased with increasing age in all regions studied, namely, the ascending, proximal aorta, and abdominal aorta. In both men and women, the greatest decreases in aortic distensibility occurred between those aged 50–59 and 60–69 years, although the decrease between these age groups was larger in women (47–61%) than in men (31–45%). These findings indicate that the decline in aortic distensibility is sex-independent, although the steepest decline occurs in women between the pre- to the postmenopausal periods. Surprisingly, however, these aortic distensibility changes were not mirrored by commensurate steep increases in PWV in the peri-menopausal period (Nethononda et al., 2015). One mechanism that may contribute to the rapid decline in aortic distensibility in females between these age groups is body weight changes that accompany menopause. Greater weight gain and an increase in the waist:hip ratio, suggesting increased abdominal fat, are seen in women compared to the increases in correspondingly aged men (Nethononda et al., 2015). Interestingly, other studies have also noted similar age-related but sex-independent PWV increases (Smulyan et al., 2001; Hickson et al., 2010). It is possible that no sex differences are observed in aortic PWV because despite distensibility decreasing rapidly after menopause, blood viscosity increases with menopause (Schillaci et al., 1998). The mathematical relationship between PWV and distensibility is demonstrated by the Bramwell-Hill equation; PWV (ρ× Distensibility)1/2, with (ρ being blood density, Nethononda et al., 2015). Given that blood viscosity increases greatly with menopause, the equation aids us in understanding why a large decrease in distensibility does not correlate with an increase in PWV of the same magnitude (Nethononda et al., 2015). However, in contrast, the Baltimore Longitudinal Study of Aging identified a steeper longitudinal increase of PWV in men compared to age-matched women (Alghatrif et al., 2013).

It is important to note that, although aortic stiffness is more severe in males than females prior to menopause, pre-menopausal women also exhibit age-dependent increases in aortic stiffness, as observed in primates (Babici et al., 2020). An aortic pressure catheter and ultrasonic diameter transducers implanted in young (7 ± 0.7 years old) and aging premenopausal female monkeys (24 ± 0.7 years old) found that the aortic pulse pressure was increased in old premenopausal monkeys (48 ± 2.7 mmHg) compared to young monkeys (33 ± 2.5 mmHg) (Babici et al., 2020). The aortic stiffness index, a function of aortic pressure and aortic strain, was increased in the old vs. young subjects in both the thoracic and abdominal aortas. Furthermore, the collagen/elastin ratio increased down the aortic tree and was consistently higher in the old premenopausal monkeys (Babici et al., 2020). Elastin and collagen showed progressively more disarray down the aortic tree (quantitation of disarray is described in the section on Mechanisms). Twice as much disarray was noted in the older group compared to the younger group in the thoracic aorta, abdominal aorta, and iliac artery (Babici et al., 2020). Elastin- and collagen-fiber disarray and breaks also increased down the aortic tree and were more marked in premenopausal monkeys. In a previous study, elastin and collagen disarray correlated better with stiffness than elastin and collagen content (Zhang et al., 2016). Studies on these structural proteins also identified sex differences in the specific characteristics of elastin and collagen. In the human abdominal aorta, elastin content decreased but the stiffness of elastin and collagen increased with age in men (Astrand et al., 2011). There was a much lesser age-related change in aortic elastin- and collagen-stiffness between young, middle-aged, and elderly women (Astrand et al., 2011). Collagen and elastin seem less affected in the female aortic wall due to the influence of sex hormones.

The mechanisms underlying age-related and regional differences in aortic stiffness are also sexually dimorphic. In non-human primate models, aging-related changes in gene expression have been shown to be sex-dependent and to involve key contributors of vascular stiffness, such as ECM composition, VSMC phenotype, cell signaling pathways, resistance to apoptosis, metabolism, protein synthesis, and transcription factors. Aging male, but not female monkeys, show downregulation of collagen type III protein expression and upregulation of collagen type VIII transcript levels. Collagen type III decreases collagen bundle size and increases vascular elasticity while collagen type VIII promotes VSMC migration into the intima (Qiu et al., 2007b). This may explain the finding that elastin stiffness increases with age in men, but not in women (Astrand et al., 2011). Similar protein and gene expression changes have also been observed in another study of a non-human primate model, in which female premenopausal animals were compared with their aged-matched male counterparts (Qiu et al., 2007a). In addition to changes in gene regulation, sex hormones are also potential contributors to sex-specific differences in age-related increases in vascular stiffness.

### Role of Sex Related Hormones

The greater increases in vascular stiffness in aged-matched men, than in women prior to menopause, is reversed, so that after menopause women show more severe increases in vascular stiffness. The difference in vascular stiffness between pre- and postmenopausal women is primarily ascribed to proposed protective roles of estrogen, which reduces increased stiffness with age. Estrogen levels rapidly decrease just before menopause and continue to fall after menopause (Moreau and Hildreth, 2014). This coincides with the timing of the accelerated age-associated vascular stiffness seen in women after menopause, when compared to age-matched men and premenopausal women. Animal studies have found that estrogen can increase elastin content, inhibit collagen deposition, and prevent abnormal VSMC proliferation and migration (Dubey et al., 2000a,b; Yoon et al., 2001). In other words, estrogen appears to counteract many of the mechanisms associated with age-dependent vascular stiffening. Furthermore, hormonal therapy with estrogen, initiated around the time of menopause, decreases arterial stiffening in postmenopausal women when compared to non-hormonal therapy-treated postmenopausal women (Moreau and Hildreth, 2014). Arterial stiffening is also associated with increased vascular tone and increased oxidative stress. Estrogen is also a potent antioxidant and prevents scavenging of nitric oxide (NO) by reactive oxygen species (Moreau and Hildreth, 2014). Thus, prolonged estrogen deficiency in postmenopausal women, by limiting availability of estrogen as an antioxidant and of NO, may contribute to an increase in vascular tone, and in susceptibility to oxidative stress. Nevertheless, it is of interest that despite the evidence for a protective role of estrogen with respect to arterial stiffness during the fertile period, epidemiological evidence suggests that there is no sudden increase in the rate of cardiovascular disease in women at the time of menopause, but this is observed rather after menopause (Woodward, 2019). Furthermore, clinical trials have found no overall cardiovascular benefit of exogenous estrogen in postmenopausal women (Boardman et al., 2015).

In mice, NO bioavailability has been directly linked to the mechanical properties of the vessel wall, corroborating that the effect of estrogen is mediated by NO. Female ovariectomized mice display increased circumferential elastic modulus in all arteries, suggesting stiffening, as well as decreased eNOS protein expression, and reduced endogenous NO production (Guo et al., 2006). Female mice lacking the endothelial NO synthase gene displayed increases in circumferential modulus in the aorta and decreased NO production in the femoral and carotid arteries (Guo et al., 2006). This supports the idea that estrogen works to reduce stiffness by stimulating NO production, which maintains structural and mechanical properties of arteries. When rodent models were given an NO synthase inhibitor, either acutely or chronically, PWV increased compared to that in controls. In fact, chronic administration showed an additional 8% increase in PWV when compared to the control experimental group (Fitch et al., 2001).

Administration of BH_4_ (tetrahydrobiopterin), a critical cofactor for NO production, increased carotid artery compliance and brachial artery flow-mediated dilatation in postmenopausal, but not premenopausal women (Moreau et al., 2012). This increase in carotid compliance was also seen when estradiol was administered to postmenopausal women (Moreau et al., 2012). However, no additional improvement was seen when BH_4_ and estradiol were co-administered (Moreau et al., 2012). Therefore, reduced BH_4_ may contribute to arterial stiffness in postmenopausal women and estrogen may increase BH_4_ bioavailability.

These varying trends between men and women significantly impact their cardiovascular disease susceptibility (Dupont et al., 2019). Heart failure with preserved ejection fraction (HFpEF) is observed twice as commonly in women than men. This increased incidence of HFpEF in women may be associated with the increased proximal aortic stiffness in post-menopausal women, when compared to men (Coutinho et al., 2013). The mean age in this study was 65 years for post-menopausal women and 67 for men (Coutinho et al., 2013). Women also exhibit more pronounced age-related increases in aortic flow impedance than men (Coutinho et al., 2013). These pathophysiological changes may have a cascade effect whereby lower aortic compliance leads to greater impedance to flow. In turn, this increases hemodynamic load on the left ventricle, resulting in an enhanced propensity for women to develop heart failure.

The enhanced arterial stiffness of older postmenopausal women, compared to their male counterparts, is an issue that warrants greater research not only to better understand the mechanisms behind this phenomenon, but also to develop more targeted and effective therapies for postmenopausal women. Isolated systolic hypertension is more common in older women and these women are less likely to achieve optimal blood pressure control than age-matched men (Dupont et al., 2019).

As mentioned previously, findings from studies of vascular aging in rodents are limited, as rodents have a shorter lifespan than monkeys or humans, and do not go through menopause. This may explain why one study of aging in rats showed similar levels of aortic stiffness at 6, 12, and 24 months of age in males and females (Mitchell et al., 2004). One rodent species that does live over 20 years, the naked mole rat, has been found to be better protected against aging-induced oxidative stress and apoptotic cell death than its shorter-living counterparts; differences that likely contribute to their exceptional longevity (Csiszar et al., 2007). This provides insight into the key roles oxidative stress may have on age-related arterial stiffness. Further studies may also be warranted of male and female naked mole rats to determine if, like human and monkeys, they also display sex-specific differences in longevity.

Sexually dimorphic longevity is observed in many mammalian species, with females living longer than males (Hagg and Jylhava, 2021). Despite differences in cardiac physiology and anatomy, rodent models have been helpful in beginning to understand mechanisms underlying sex-specific differences in vascular aging (Blenck et al., 2016). However, monkey models are more applicable to humans than rodents because they live longer and have a menstrual cycle. But with the availability of non-human primate models, more research is required to fully elucidate the key drivers of sex-specific differences in vascular aging.

### Vascular Stiffness in Prepubescent Years

Sex differences in arterial stiffening have been found even in prepubescent human subjects and may be attributed to both sex steroids and intrinsic differences. Prepubescent girls, for example, have less compliant arteries and higher central and peripheral PWV than their male counterparts (Ahimastos et al., 2003). However, after puberty, central PWV falls in females but increases in male (Ahimastos et al., 2003). Interestingly, prepubescent and postmenopausal arterial stiffness are both likely due to the same mechanism, namely, low levels of sex steroids.

## Diseases States

### Hypertension

Aortic stiffness and arterial pressure are strongly correlated in hypertension with vascular stiffness being both a cause and a consequence of hypertension (Humphrey et al., 2016). High blood pressure may cause vascular damage and elastin fragmentation, leading to increased stiffness. On the other hand, aortic stiffness widens pulse pressure which affects systolic blood pressure. Hypertension and aging may have an additive effect evidenced by elderly hypertensive patients having stiffer arteries than age-matched normotensive patients (Verwoert et al., 2014). However, these differences may be attributed to hypertension, rather than intrinsic vascular changes associated with increased stiffness (Bavishi et al., 2016).

**FIGURE 9:**
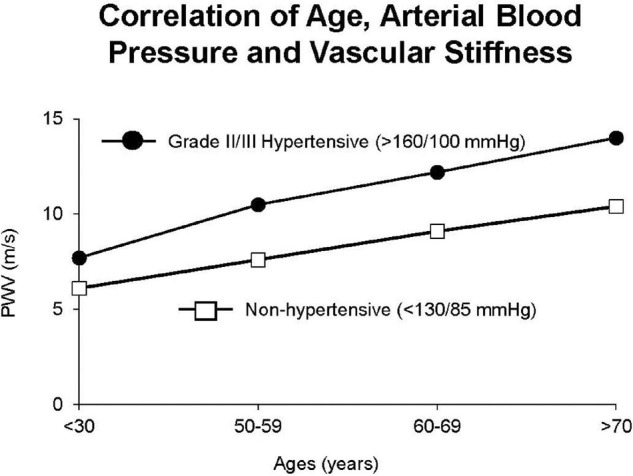
Interaction between hypertension and arterial stiffness in patients. There were similar linear relationships in hypertensive patients vs. non-hypertensive patients, but with stiffness greater in hypertensive patients at all ages (reprint from Safar et al., 2018).

Vascular stiffness is linearly related to age both in normotensive and severely hypertensive subjects (Figure 9; Safar et al., 2018). Interestingly the slope of these linear relationships is not that different (Figure 9); arterial stiffness rising in normotensive people almost as much as in those who are hypertensive. Aortic stiffness is also increased in spontaneously hypertensive rats, even at a young age, but much more in older rats (Sehgel et al., 2013, 2015a,b; Figure 8). As discussed above, elastin breakdown, due to matrix metallopeptidases and serum elastase, is a major mediator of increased vascular stiffness (4). Serum MMP-9 and MMP-2 levels, and serum elastase activity, which degrade elastin degradation and increase aortic and brachial PWV, are increased in subjects with isolated systolic hypertension (Yasmin et al., 2005).

Isolated systolic hypertension, defined as systolic blood pressure > 140 mmHg and diastolic blood pressure < 90 mm Hg (Mancia et al., 2013), is the predominant form of hypertension in the elderly, and is associated with increased arterial stiffness (Franklin et al., 1997; Yasmin et al., 2005; Bavishi et al., 2016). Interestingly, in older patients, systolic arterial pressure continues to increase along with aortic stiffness, but diastolic hypertension declines, further demonstrating the important relationship between systolic arterial hypertension and aortic stiffness (Franklin et al., 1997). Systolic-diastolic hypertension due to elevation of both systolic and diastolic arterial pressures is less common in older adults (Tsimploulis et al., 2017), but is associated with an increased incidence of heart failure and cardiovascular mortality (Tsimploulis et al., 2017). Also, pre-eclampsia, which induces hypertension during pregnancy, is associated with increased vascular stiffness (Hausvater et al., 2012).

Salt intake, a key mechanism mediating hypertension, is positively correlated with carotid-femoral P; the slope of the linear regression line linking these two parameters was steeper in women than in men (0.0199 ± 0.0045 vs. 0.0326 ± 0.0052 m/s per gram of salt, respectively, *P* < 0.05). However, after adjustment in the data of outliers, the association remained significant only in men (Baldo et al., 2019).

Calcium deposition in the aorta is another mechanism mediating the increase in arterial stiffness in hypertension (Guo et al., 2017). Interestingly, it was most pronounced in subjects who were resistant to anti-hypertensive therapy, suggesting that arterial stiffness not only contributes to isolated systolic hypertension development, but may also be involved in resistance to hypertension treatment (Mceniery et al., 2009).

Another important mechanism of aortic stiffness in hypertension is vascular smooth muscle stiffness (Sehgel et al., 2013, 2015a,b). Changes to the intrinsic stiffness of VSMCs and to their adhesion properties are observed in hypertension. Using atomic force microscopy and a reconstituted aortic tissue model, it was found that spontaneously hypertensive rats had increased aortic VSMC stiffness as well as different temporal oscillations in VSMC stiffness compared to normotensive control rats (Sehgel et al., 2013). In a later study in which VSMC stiffness and adhesions to the ECM were also found to be increased in hypertensive rats (Sehgel et al., 2015a), hypertension did not increase the amount of collagen in the thoracic aorta, suggesting that increased vascular stiffness associated with hypertension is likely not exclusively mediated by altered collagen and elastin content, but by increased VSMC stiffness and adhesion (Sehgel et al., 2015a). VSMC changes with aging are augmented when hypertension is superimposed on aging.

### Atherosclerosis

Atherosclerosis, which is more common with aging, is a chronic inflammatory disease in which atheromatous plaques form, resulting in arterial narrowing. Many studies, such as the Rotterdam study (Van Popele et al., 2001), show that stiffness of the aorta increases with plaque burden and conclude that arterial stiffness is strongly associated with atherosclerosis. However, these conclusions must be tempered by the fact that increased vascular stiffness is also a feature of aging in the absence of atherosclerosis (Sun, 2015). Atherosclerosis can occur at an earlier age in a condition known as Pediatric atherosclerosis (Wilson, 2000). Also, as discussed above the autosomal recessive premature aging disorder, Hutchison-Gilford Progeria syndrome, is characterized by precocious atherosclerosis and stiffening of the arteries, which cause early death in affected individuals (Keay et al., 1955). Consistent with the arterial stiffness found in these children, their carotid-femoral PWV is also markedly increased (Gerhard-Herman et al., 2012; Gordon et al., 2012). One way to address the question of mechanisms of atherosclerosis in a younger population is to examine atherosclerosis mechanisms in animal models that develop disease at a young age. Our laboratory has begun to study aortic stiffness in Watanabe rabbits, an animal model of atherosclerosis (Aliev and Burnstock, 1998). Our preliminary data suggest that aortic stiffness is increased even in relatively young Watanabe rabbits, as compared to aged-matched New Zealand White rabbit controls. In hypercholesterolemia Kurosawa and Kusanagi rabbits, PWV reflecting the atherosclerotic regions found that vascular stiffness was increased more in these regions and more in the abdominal vs. the thoracic aorta (Katsuda et al., 2014).

Many different mechanisms have been implicated in the stiffening of arteries associated with atherosclerosis. Hypercholesterolemia is most commonly implicated in the pathogenesis of atherosclerosis in humans and animal models, ranging from rodents to rabbits, pigs and monkeys (Linton et al., 2000; Getz and Reardon, 2012). Extracellular matrix proteins are also involved in the development of increased vascular stiffness in atherosclerosis. Elastin degradation is increased by the build-up of atheromatous plaques. Non-atherosclerotic arteries contain MMP-2 as well as inhibitors of MMP, such as TIMP 1 and 2 (Galis et al., 1994). In contrast, atheromatous plaques also contain macrophages that secrete MMP-1, MMP-9, and MMP-3, smooth muscle cells, lymphocytes, and endothelium (Galis et al., 1994). Based on SDS-PAGE zymography, plaques have been found to contain activated forms of MMP-2 and MMP-9 (Galis et al., 1994). In addition, patients with hypercholesterolemia exhibit more circulating CD31^+^/CD42^–^ microparticles, less endothelial progenitors (EPCs), and have stiffer aortae than controls. The ratio of CD31^+^/CD42^–^ microparticles to EPCs was found to be directly associated with arterial PWV (aPWV) (Pirro et al., 2006). This suggests that hypercholesterolemia contributes to large artery stiffness by increasing microparticle release and by reducing the number of circulating EPCs (Pirro et al., 2006). In addition, the extracellular matrix protein, fibrillin-1, has been found to modulate large-artery stiffness and pulse pressure (Medley et al., 2002).

Elevated oxidative stress also plays a role in increased arterial stiffening in patients with atherosclerosis. A study examining patients with peripheral arterial disease found an independent association of aPWV with serum levels of osteopontin and oxidized low-density lipoprotein, which are involved in oxidative stress, thus supporting the role for oxidative stress in mediating arterial stiffness in patients with atherosclerosis (Zagura et al., 2012).

Intimal arterial calcification within atherosclerotic plaques may also be responsible for increased vascular stiffness (Mackey et al., 2007). A study in the Twins United Kingdom population suggests that it is the propensity of plaques to calcify rather than the amount of plaque that determines arterial stiffness (Cecelja et al., 2013). Aortic stiffness was correlated with calcified plaques in the carotid and femoral arteries detected by ultrasound and with total aortic calcification measured by computed tomography (Cecelja et al., 2013).

### Diabetes

Diabetes predisposes to cardiovascular disease and accelerated arterial stiffness. The magnitude of the effect of diabetes on central stiffness has been compared to the equivalent of 6–15 years of chronological aging on vessels (Cameron and Cruickshank, 2007; Loehr et al., 2016). It has been suggested that vascular stiffness in diabetic patients may be attributed more to the role of diabetes and metabolism, than to aging, *per se* (Cameron et al., 2003). One major metabolic mechanism is the non-enzymatic advanced glycation of proteins observed in diabetes. The accelerated production of advanced glycation end-products (AGEs) is implicated in diabetes-associated increasing stiffness. AGEs form in hyperglycemic environments, accumulate in the vessel wall, and form cross-links with collagen and elastin fibers, decreasing arterial wall distensibility (Goldin et al., 2006). The incidence of atherosclerosis is also increased in diabetic patients (Poznyak et al., 2020) and, thus, increased vascular stiffening due to accelerated atherosclerosis also contributes to the increase vascular stiffness in diabetic patients (Stehouwer et al., 2008; Prenner and Chirinos, 2015). Patients with type 2 diabetes also displayed endothelial dysfunction and a reduced contractile response to endothelin-1, suggesting these mechanisms factor into the development of vascular stiffness, due to the role of vasoconstriction in mediating vascular stiffness (Rizzoni et al., 2001). For example, endothelium-dependent dilation has shown to be abnormal in patients with type 2 diabetes attributed mainly to dyslipidemia (Schofield et al., 2002).

It has been shown that vascular stiffness increases as glucose tolerance deteriorates. Impaired glucose metabolism and type 2 diabetes (DM-2) are associated with decreased total systemic arterial compliance and increased aortic augmentation index, indicating increased central artery stiffness (Schram et al., 2004). Central artery stiffness is greater and carotid-femoral transit time is decreased in patients with DM-2 (Schram et al., 2004). It has also been shown that stiffness of the peripheral arteries increases with deteriorating glucose tolerance (Henry et al., 2003). Together, these studies suggest that stiffness due to impaired glucose metabolism and DM-2 are worse in peripheral than central arteries (Henry et al., 2003; Schram et al., 2004). Interestingly, in children with type 1 diabetes, especially males, stiffness of peripheral arteries is more common than of central arteries (Urbina et al., 2010).

As noted throughout this review, an important mechanism mediating increased vascular stiffness is increased oxidative stress (Giacco and Brownlee, 2010; Pasupuleti et al., 2020). It is well known that diabetes leads to increased oxidative stress, involving mitochondrial superoxide overproduction in the vasculature and in in the myocardium (Giacco and Brownlee, 2010; Pasupuleti et al., 2020). It is also recognized that increased intracellular reactive oxygen species cause defective angiogenesis in response to ischemia, and activate a number of proinflammatory pathways in diabetes and mediate the atherosclerosis and cardiomyopathy associated with diabetes (Giacco and Brownlee, 2010; Pasupuleti et al., 2020).

Even patients with prediabetes experience increased arterial stiffness. In one study, diabetes was associated with higher aortic PWV and prediabetes was associated with higher brachial-ankle PWV, a measure of composite stiffness (Loehr et al., 2016). Similarly, it has been shown that higher baPWV is associated with an increased risk of developing diabetes and that arterial stiffness may precede the increase in fasting blood glucose (Zheng et al., 2020). Arterial stiffness is also increased in patients with impaired fasting glucose but no other cardiovascular complications (Rerkpattanapipat et al., 2009). That study found that total vascular stiffness, but not thoracic aortic stiffness, is increased in patients with impaired fasting glucose compared to control subjects (Rerkpattanapipat et al., 2009).

## Conclusion

One of the most important effects of aging on the cardiovascular system is a progressive increase in vascular stiffness. Understanding the extent to which vascular stiffness increases with aging and the mechanisms involved are important, since vascular stiffness is a critical factor in mediating the adverse effects of most cardiovascular diseases, including atherosclerosis, hypertension and diabetes. Many prior studies are limited in defining changes in vascular stiffness down the aortic tree, because only one section of the aorta was studied. We found that abdominal aortic stiffness is greater than thoracic aortic stiffness. However, this topic warrants further investigation as there are major sex differences. In men vascular stiffness increases progressively from young adulthood to old age. In women vascular stiffness increases, but to a lesser extent up to menopause, and then increases at a rate exceeding that for males after menopause. Less data are available on sex differences in animals, since the most commonly studied species are rodents, where females do not go through menopause, Their relevance for understanding human disease, therefore, is limited. Studies of sex differences in the changes in vascular stiffness associated with age are best carried out in humans without associated cardiovascular diseases, and in non-human primates that live over 30 years and, like human females, go through menopause. Several mechanisms mediate the protection in females, with the most significant one being the female hormone, estrogen, which is present up to menopause and then declines. Other important mechanisms of increased vascular stiffness include changes in the extracellular matrix, with increases in vascular collagen and decreases in vascular elastin. It is also known that calcium deposition and endothelial dysfunction in the vessels contribute to increased vascular stiffness. Less well studied mechanisms may also contribute, such as collagen and elastin disarray, and increased vascular smooth muscle cell stiffness and numbers. Additional insights come from studies in populations with an extended lifespan that live in areas known as “Blue Zones.” People in these areas maintain a healthy diet and daily exercise and have lesser increases in vascular stiffness with age. Understanding how these environmental factors influence the progression of vascular stiffness may provide critical insights into retarding its progression and, thereby reducing cardiovascular disease.

## Future Directions

As elucidated in this review, considerable progress has been made in understanding the role of vascular stiffness in normal biology and aging as well as in mediating changes in disease states. However, considerably more work needs to be done in this field. Differences in stiffness down the aortic tree in regional arteries and veins and arterial resistance vessels requires further research, since most prior work only examined the aorta. More work is also required to understand the divergent effects of increased aortic stiffness in males and females, and the role of sex hormones in mediating those differences. There is much more to be learned about the molecular mechanisms mediating changes in arterial stiffness. There is also much more clarification needed to understand the relationship between disease states in affecting changes in vascular stiffness, and conversely, how vascular stiffness mediates disease states. An important example is atherosclerosis, where it is needed to understand the extent to which changes in vascular function in atherosclerosis are due to changes in vascular stiffness vs. changes due to atheroma.

## Author Contributions

SV: conceptualization. SV, JZ, CV, KM, and DV: writing—original draft. SV, JZ, CV, KM, RG, and DV: writing—review and editing. All authors contributed to the article and approved the submitted version.

## Conflict of Interest

The authors declare that the research was conducted in the absence of any commercial or financial relationships that could be construed as a potential conflict of interest.

## Publisher’s Note

All claims expressed in this article are solely those of the authors and do not necessarily represent those of their affiliated organizations, or those of the publisher, the editors and the reviewers. Any product that may be evaluated in this article, or claim that may be made by its manufacturer, is not guaranteed or endorsed by the publisher.

## References

[B1] Ahimastos A. A. Formosa M. Dart A. M. Kingwell B. A. (2003). Gender differences in large artery stiffness pre- and post puberty. *J. Clin. Endocrinol. Metab.* 88 5375–5380. 10.1210/jc.2003-030722 14602776

[B2] Ahmet I. Tae H. J. De Cabo R. Lakatta E. G. Talan M. I. (2011). Effects of calorie restriction on cardioprotection and cardiovascular health. *J. Mol. Cell Cardiol.* 51 263–271. 10.1016/j.yjmcc.2011.04.015 21586294 PMC3138119

[B3] Alghatrif M. Strait J. B. Morrell C. H. Canepa M. Wright J. Elango P. (2013). Longitudinal trajectories of arterial stiffness and the role of blood pressure: the Baltimore Longitudinal Study of Aging. *Hypertension* 62 934–941. 10.1161/HYPERTENSIONAHA.113.01445 24001897 PMC3880832

[B4] Aliev G. Burnstock G. (1998). Watanabe rabbits with heritable hypercholesterolaemia: a model of atherosclerosis. *Histol. Histopathol.* 13 797–817. 9690137 10.14670/HH-13.797

[B5] Anderson T. J. (2006). Arterial stiffness or endothelial dysfunction as a surrogate marker of vascular risk. *Can. J. Cardiol.* 22 (Suppl. B), 72B–80B. 10.1016/s0828-282x(06)70990-4 PMC2780833 16498516

[B6] Aronson D. (2003). Cross-linking of glycated collagen in the pathogenesis of arterial and myocardial stiffening of aging and diabetes. *J. Hypertens.* 21 3–12. 12544424 10.1097/00004872-200301000-00002

[B7] Astrand H. Stalhand J. Karlsson J. Karlsson M. Sonesson B. Lanne T. (2011). In vivo estimation of the contribution of elastin and collagen to the mechanical properties in the human abdominal aorta: effect of age and sex. *J. Appl. Physiol.* 110 176–187. 10.1152/japplphysiol.00579.2010 21071586

[B8] Atanasova M. Dimitrova A. Ruseva B. Stoyanova A. Georgieva M. Konova E. (2012). “Quantification of elastin, collagen and advanced glycation end products as functions of age and hypertension,” in *Agriculture and Biological Sciences, Senescence*, ed. Nagata T. (London: InTechOpen).

[B9] Babici D. Kudej R. K. Mcnulty T. Zhang J. Oydanich M. Berkman T. (2020). Mechanisms of increased vascular stiffness down the aortic tree in aging, premenopausal female monkeys. *Am. J. Physiol. Heart Circ. Physiol.* 319 H222–H234. 10.1152/ajpheart.00153.2020 32530752 PMC7474445

[B10] Baldo M. P. Brant L. C. C. Cunha R. S. Molina M. Griep R. H. Barreto S. M. (2019). The association between salt intake and arterial stiffness is influenced by a sex-specific mediating effect through blood pressure in normotensive adults: the ELSA-Brasil study. *J. Clin. Hypertens.* 21 1771–1779. 10.1111/jch.13728 31742882 PMC8030347

[B11] Barton M. Cosentino F. Brandes R. P. Moreau P. Shaw S. Lüscher T. F. (1997). Anatomic heterogeneity of vascular aging. *Hypertension* 30 817–824. 10.1161/01.hyp.30.4.817 9336378

[B12] Bavishi C. Goel S. Messerli F. H. (2016). Isolated systolic hypertension: an update after SPRINT. *Am. J. Med.* 129 1251–1258. 10.1016/j.amjmed.2016.08.032 27639873

[B13] Benetos A. Laurent S. Hoeks A. P. Boutouyrie P. H. Safar M. E. (1993). Arterial alterations with aging and high blood pressure. A noninvasive study of carotid and femoral arteries. *Arterioscler. Thromb.* 13 90–97. 8422344 10.1161/01.atv.13.1.90

[B14] Blaha M. J. Budoff M. J. Rivera J. J. Katz R. O’leary D. H. Polak J. F. (2009). Relationship of carotid distensibility and thoracic aorta calcification: multi-ethnic study of atherosclerosis. *Hypertension* 54 1408–1415. 10.1161/HYPERTENSIONAHA.109.138396 19805639 PMC4118641

[B15] Blenck C. L. Harvey P. A. Reckelhoff J. F. Leinwand L. A. (2016). The importance of biological sex and estrogen in rodent models of cardiovascular health and disease. *Circ. Res.* 118 1294–1312. 27081111 10.1161/CIRCRESAHA.116.307509 PMC4834858

[B16] Boardman H. M. Hartley L. Eisinga A. Main C. Roque I. Figuls M. (2015). Hormone therapy for preventing cardiovascular disease in post-menopausal women. *Cochrane Database Syst. Rev.* 10:CD002229. 10.1002/14651858.CD002229.pub4 PMC10183715 25754617

[B17] Boutouyrie P. Laurent S. Girerd X. Benetos A. Lacolley P. Abergel E. (1995). Common carotid artery stiffness and patterns of left ventricular hypertrophy in hypertensive patients. *Hypertension* 25 651–659. 10.1161/01.hyp.25.4.651 7721411

[B18] Briones A. M. Arribas S. M. Salaices M. (2010). Role of extracellular matrix in vascular remodeling of hypertension. *Curr. Opin. Nephrol. Hypertens.* 19 187–194. 10.1097/mnh.0b013e328335eec9 20040870

[B19] Buettner D. Skemp S. (2016). Blue zones: lessons from the world’s longest lived. *Am. J. Lifestyle Med.* 10 318–321. 10.1177/1559827616637066 30202288 PMC6125071

[B20] Byon C. H. Javed A. Dai Q. Kappes J. C. Clemens T. L. Darley-Usmar V. M. (2008). Oxidative stress induces vascular calcification through modulation of the osteogenic transcription factor Runx2 by AKT signaling^∗^. *J. Biol. Chem.* 283 15319–15327. 10.1074/jbc.M800021200 18378684 PMC2397455

[B21] Cameron J. D. Bulpitt C. J. Pinto E. S. Rajkumar C. (2003). The aging of elastic and muscular arteries: a comparison of diabetic and nondiabetic subjects. *Diabetes Care* 26 2133–2138. 10.2337/diacare.26.7.2133 12832325

[B22] Cameron J. D. Cruickshank J. K. (2007). Glucose, insulin, diabetes and mechanisms of arterial dysfunction. *Clin. Exp. Pharmacol. Physiol.* 34 677–682. 17581229 10.1111/j.1440-1681.2007.04659.x

[B23] Cecelja M. Hussain T. Greil G. Botnar R. Preston R. Moayyeri A. (2013). Multimodality imaging of subclinical aortic atherosclerosis: relation of aortic stiffness to calcification and plaque in female twins. *Hypertension* 61 609–614. 10.1161/HYPERTENSIONAHA.111.00024 23339166

[B24] Chamiot-Clerc P. Renaud J. F. Safar M. E. (2001). Pulse pressure, aortic reactivity, and endothelium dysfunction in old hypertensive rats. *Hypertension* 37 313–321. 11230291 10.1161/01.hyp.37.2.313

[B25] Chi C. Li D. J. Jiang Y. J. Tong J. Fu H. Wu Y. H. (2019). Vascular smooth muscle cell senescence and age-related diseases: State of the art. *Biochim. Biophys. Acta Mol. Basis Dis.* 1865 1810–1821. 10.1016/j.bbadis.2018.08.015 31109451

[B26] Collins J. A. Munoz J.-V. Patel T. R. Loukas M. Tubbs R. S. (2014). The anatomy of the aging aorta. *Clin. Anat.* 27 463–466. 10.1002/ca.22384 24523152

[B27] Coutinho T. Borlaug B. A. Pellikka P. A. Turner S. T. Kullo I. J. (2013). Sex differences in arterial stiffness and ventricular-arterial interactions. *J. Am. Coll. Cardiol.* 61 96–103. 23122799 10.1016/j.jacc.2012.08.997 PMC3773521

[B28] Csiszar A. Labinskyy N. Orosz Z. Xiangmin Z. Buffenstein R. Ungvari Z. (2007). Vascular aging in the longest-living rodent, the naked mole rat. *Am. J. Physiol. Heart Circ. Physiol.* 293 H919–H927. 17468332 10.1152/ajpheart.01287.2006

[B29] Cuomo F. Roccabianca S. Dillon-Murphy D. Xiao N. Humphrey J. D. Figueroa C. A. (2017). Effects of age-associated regional changes in aortic stiffness on human hemodynamics revealed by computational modeling. *PLoS One* 12:e0173177. 10.1371/journal.pone.0173177 28253335 PMC5333881

[B30] Dammann P. Burda H. (2006). Sexual activity and reproduction delay ageing in a mammal. *Curr. Biol.* 16 R117–R118. 10.1016/j.cub.2006.02.012 16488857

[B31] Devos D. G. Rietzschel E. Heyse C. Vandemaele P. Van Bortel L. Babin D. (2015). MR pulse wave velocity increases with age faster in the thoracic aorta than in the abdominal aorta. *J. Magn. Reson. Imaging* 41 765–772. 10.1002/jmri.24592 24615998

[B32] Donato A. J. Machin D. R. Lesniewski L. A. (2018). Mechanisms of dysfunction in the aging vasculature and role in age-related disease. *Circ. Res.* 123 825–848. 10.1161/circresaha.118.312563 30355078 PMC6207260

[B33] Dubey R. K. Gillespie D. G. Zacharia L. C. Rosselli M. Korzekwa K. R. Fingerle J. (2000a). Methoxyestradiols mediate the antimitogenic effects of estradiol on vascular smooth muscle cells via estrogen receptor-independent mechanisms. *Biochem. Biophys. Res. Commun.* 278 27–33. 10.1006/bbrc.2000.3755 11071850

[B34] Dubey R. K. Jackson E. K. Gillespie D. G. Zacharia L. C. Imthurn B. Keller P. J. (2000b). Clinically used estrogens differentially inhibit human aortic smooth muscle cell growth and mitogen-activated protein kinase activity. *Arterioscler. Thromb. Vasc. Biol.* 20 964–972. 10.1161/01.atv.20.4.964 10764660

[B35] Dupont J. J. Kenney R. M. Patel A. R. Jaffe I. Z. (2019). Sex differences in mechanisms of arterial stiffness. *Br. J. Pharmacol.* 176 4208–4225. 10.1111/bph.14624 30767200 PMC6877796

[B36] Faber M. Oller-Hou G. (1952). The human aorta. V. Collagen and elastin in the normal and hypertensive aorta. *Acta Pathol. Microbiol. Scand.* 31 377–382. 12985344

[B37] Farrar D. J. Bond M. G. Sawyer J. K. Green H. D. (1984). Pulse wave velocity and morphological changes associated with early atherosclerosis progression in the aortas of cynomolgus monkeys. *Cardiovasc. Res.* 18 107–118. 10.1093/cvr/18.2.107 6697337

[B38] Fischer G. M. Llaurado J. G. (1966). Collagen and elastin content in canine arteries selected from functionally different vascular beds. *Circ. Res.* 19 394–399. 10.1161/01.res.19.2.394 5914851

[B39] Fitch R. M. Vergona R. Sullivan M. E. Wang Y.-X. (2001). Nitric oxide synthase inhibition increases aortic stiffness measured by pulse wave velocity in rats. *Cardiovasc. Res.* 51 351–358. 10.1016/s0008-6363(01)00299-1 11470475

[B40] Fleenor B. S. Marshall K. D. Durrant J. R. Lesniewski L. A. Seals D. R. (2010). Arterial stiffening with ageing is associated with transforming growth factor-β1-related changes in adventitial collagen: reversal by aerobic exercise. *J. Physiol.* 588 3971–3982. 10.1113/jphysiol.2010.194753 20807791 PMC3000586

[B41] Fleenor B. S. Marshall K. D. Rippe C. Seals D. R. (2012). Replicative aging induces endothelial to mesenchymal transition in human aortic endothelial cells: potential role of inflammation. *J. Vasc. Res.* 49 59–64. 10.1159/000329681 21985896 PMC3214888

[B42] Fornieri C. Quaglino D. Jr. Mori G. (1992). Role of the extracellular matrix in age-related modifications of the rat aorta. Ultrastructural, morphometric, and enzymatic evaluations. *Arterioscler. Thromb.* 12 1008–1016. 10.1161/01.atv.12.9.1008 1356019

[B43] Fornieri C. Taparelli F. Quaglino D. Jr. Contri M. B. Davidson J. M. Algeri S. (1999). The effect of caloric restriction on the aortic tissue of aging rats. *Connect. Tissue Res.* 40 131–143. 10.3109/03008209909029109 10761638

[B44] Franklin S. S. Gustin W. T. Wong N. D. Larson M. G. Weber M. A. Kannel W. B. (1997). Hemodynamic patterns of age-related changes in blood pressure. The Framingham Heart Study. *Circulation* 96 308–315. 10.1161/01.cir.96.1.308 9236450

[B45] Furchgott R. F. Zawadzki J. V. (1980). The obligatory role of endothelial cells in the relaxation of arterial smooth muscle by acetylcholine. *Nature* 288 373–376. 10.1038/288373a0 6253831

[B46] Galis Z. S. Sukhova G. K. Lark M. W. Libby P. (1994). Increased expression of matrix metalloproteinases and matrix degrading activity in vulnerable regions of human atherosclerotic plaques. *J. Clin. Invest.* 94 2493–2503. 10.1172/JCI117619 7989608 PMC330083

[B47] Gerhard-Herman M. Smoot L. B. Wake N. Kieran M. W. Kleinman M. E. Miller D. T. (2012). Mechanisms of premature vascular aging in children with Hutchinson-Gilford progeria syndrome. *Hypertension* 59 92–97. 22083160 10.1161/HYPERTENSIONAHA.111.180919 PMC3248242

[B48] Getz G. S. Reardon C. A. (2012). Animal models of atherosclerosis. *Arterioscler. Thromb. Vasc. Biol.* 32 1104–1115. 22383700 10.1161/ATVBAHA.111.237693 PMC3331926

[B49] Giacco F. Brownlee M. (2010). Oxidative stress and diabetic complications. *Circ. Res.* 107 1058–1070. 21030723 10.1161/CIRCRESAHA.110.223545 PMC2996922

[B50] Goldin A. Beckman J. A. Schmidt A. M. Creager M. A. (2006). Advanced glycation end products. *Circulation* 114 597–605. 16894049 10.1161/CIRCULATIONAHA.106.621854

[B51] Gordon L. B. Harten I. A. Patti M. E. Lichtenstein A. H. (2005). Reduced adiponectin and HDL cholesterol without elevated C-reactive protein: clues to the biology of premature atherosclerosis in Hutchinson-Gilford Progeria Syndrome. *J. Pediatr.* 146 336–341. 10.1016/j.jpeds.2004.10.064 15756215

[B52] Gordon L. B. Kleinman M. E. Miller D. T. Neuberg D. S. Giobbie-Hurder A. Gerhard-Herman M. (2012). Clinical trial of a farnesyltransferase inhibitor in children with Hutchinson-Gilford progeria syndrome. *Proc. Natl. Acad. Sci. U.S.A.* 109 16666–16671. 23012407 10.1073/pnas.1202529109 PMC3478615

[B53] Greenberg S. R. (1986). The association of medial collagenous tissue with atheroma formation in the aging human aorta as revealed by a special technique. *Histol. Histopathol.* 1 323–326. 2980126

[B54] Greenwald S. E. (2007). Ageing of the conduit arteries. *J. Pathol.* 211 157–172. 10.1002/path.2101 17200940

[B55] Grimes K. M. Reddy A. K. Lindsey M. L. Buffenstein R. (2014). And the beat goes on: maintained cardiovascular function during aging in the longest-lived rodent, the naked mole-rat. *Am. J. Physiol. Heart Circ. Physiol.* 307 H284–H291. 10.1152/ajpheart.00305.2014 24906918 PMC4121653

[B56] Guo J. Fujiyoshi A. Willcox B. Choo J. Vishnu A. Hisamatsu T. (2017). Increased aortic calcification is associated with arterial stiffness progression in multiethnic middle-aged men. *Hypertension* 69 102–108. 10.1161/HYPERTENSIONAHA.116.08459 27821619 PMC5145727

[B57] Guo X. Lu X. Ren H. Levin E. R. Kassab G. S. (2006). Estrogen modulates the mechanical homeostasis of mouse arterial vessels through nitric oxide. *Am. J. Physiol. Heart Circ. Physiol.* 290 H1788–H1797. 10.1152/ajpheart.01070.2005 16306215

[B58] Hager A. Kaemmerer H. Rapp-Bernhardt U. Blücher S. Rapp K. Bernhardt T. M. (2002). Diameters of the thoracic aorta throughout life as measured with helical computed tomography. *J. Thorac. Cardiovasc. Surg.* 123 1060–1066. 10.1067/mtc.2002.122310 12063451

[B59] Hagg S. Jylhava J. (2021). Sex differences in biological aging with a focus on human studies. *eLife* 10:e63425. 10.7554/eLife.63425 PMC8118651 33982659

[B60] Haskett D. Johnson G. Zhou A. Utzinger U. Vande Geest J. (2010). Microstructural and biomechanical alterations of the human aorta as a function of age and location. *Biomech. Model. Mechanobiol.* 9 725–736. 10.1007/s10237-010-0209-7 20354753

[B61] Hausvater A. Giannone T. Sandoval Y. H. Doonan R. J. Antonopoulos C. N. Matsoukis I. L. (2012). The association between preeclampsia and arterial stiffness. *J. Hypertens.* 30 17–33. 22134391 10.1097/HJH.0b013e32834e4b0f

[B62] Henry R. M. A. Kostense P. J. Spijkerman A. M. W. Dekker J. M. Nijpels G. Heine R. J. (2003). Arterial stiffness increases with deteriorating glucose tolerance status. *Circulation* 107 2089–2095. 10.1161/01.cir.0000065222.34933.fc 12695300

[B63] Hickson S. S. Butlin M. Graves M. Taviani V. Avolio A. P. Mceniery C. M. (2010). The relationship of age with regional aortic stiffness and diameter. *JACC Cardiovasc Imaging* 3 1247–1255. 10.1016/j.jcmg.2010.09.016 21163453

[B64] Hofmann Bowman M. Wilk J. Heydemann A. Kim G. Rehman J. Lodato J. A. (2010). S100A12 mediates aortic wall remodeling and aortic aneurysm. *Circ. Res.* 106 145–154. 10.1161/CIRCRESAHA.109.209486 19875725 PMC2878187

[B65] Hornebeck W. Partridge S. M. (1975). Conformational changes in fibrous elastin due to calcium ions. *Eur. J. Biochem.* 51 73–78. 10.1111/j.1432-1033.1975.tb03908.x 1122917

[B66] Hosoda Y. Kawano K. Yamasawa F. Ishii T. Shibata T. Inayama S. (1984). Age-dependent changes of collagen and elastin content in human aorta and pulmonary artery. *Angiology* 35 615–621. 10.1177/000331978403501001 6497045

[B67] Huang Y. Mark Jacquez G. (2017). Identification of a blue zone in a typical chinese longevity region. *Int. J. Environ. Res. Public Health* 14:571. 10.3390/ijerph14060571 28555035 PMC5486257

[B68] Humphrey J. D. Harrison D. G. Figueroa C. A. Lacolley P. Laurent S. (2016). Central artery stiffness in hypertension and aging. *Circ. Res.* 118 379–381. 10.1161/circresaha.115.307722 26846637 PMC4745997

[B69] Iribarren C. Sidney S. Sternfeld B. Browner W. S. (2000). Calcification of the aortic archrisk factors and association with coronary heart disease, stroke, and peripheral vascular disease. *JAMA* 283 2810–2815. 10.1001/jama.283.21.2810 10838649

[B70] Jaminon A. Reesink K. Kroon A. Schurgers L. (2019). The role of vascular smooth muscle cells in arterial remodeling: focus on calcification-related processes. *Int. J. Mol. Sci.* 20:5694. 10.3390/ijms20225694 31739395 PMC6888164

[B71] Johnson R. C. Leopold J. A. Loscalzo J. (2006). Vascular calcification: pathobiological mechanisms and clinical implications. *Circ. Res.* 99 1044–1059. 10.1161/01.res.0000249379.55535.21 17095733

[B72] Kanabrocki E. L. Fels I. G. Kaplan E. (1960). Calcium, cholesterol, and collagen levels in human aortas. *J. Gerontol.* 15 383–387. 13751105 10.1093/geronj/15.4.383

[B73] Katsuda S.-I. Takazawa K. Miyake M. Kobayashi D. Kusanagi M. Hazama A. (2014). Local pulse wave velocity directly reflects increased arterial stiffness in a restricted aortic region with progression of atherosclerotic lesions. *Hypertens. Res.* 37 892–900. 10.1038/hr.2014.96 25007764

[B74] Kawasaki T. Sasayama S. Yagi S. Asakawa T. Hirai T. (1987). Non-invasive assessment of the age related changes in stiffness of major branches of the human arteries. *Cardiovasc. Res.* 21 678–687. 10.1093/cvr/21.9.678 3328650

[B75] Keay A. J. Oliver M. F. Boyd G. S. (1955). Progeria and atherosclerosis. *Arch. Dis. Child.* 30 410–414. 10.1136/adc.30.153.410 13269188 PMC2011807

[B76] Kohn J. C. Lampi M. C. Reinhart-King C. A. (2015). Age-related vascular stiffening: causes and consequences. *Front. Genet.* 6:112. 10.3389/fgene.2015.00112 PMC4396535 25926844

[B77] Komutrattananont P. Mahakkanukrauh P. Das S. (2019). Morphology of the human aorta and age-related changes: anatomical facts. *Anat. Cell Biol.* 52 109–114. 10.5115/acb.2019.52.2.109 31338225 PMC6624342

[B78] Lacolley P. Regnault V. Nicoletti A. Li Z. Michel J. B. (2012). The vascular smooth muscle cell in arterial pathology: a cell that can take on multiple roles. *Cardiovasc. Res.* 95 194–204. 22467316 10.1093/cvr/cvs135

[B79] Larocca T. J. Gioscia-Ryan R. A. Hearon C. M. Seals D. R. (2013). The autophagy enhancer spermidine reverses arterial aging. *Mech. Ageing Dev.* 134 314–320. 10.1016/j.mad.2013.04.004 23612189 PMC3700669

[B80] Larocca T. J. Henson G. D. Thorburn A. Sindler A. L. Pierce G. L. Seals D. R. (2012). Translational evidence that impaired autophagy contributes to arterial ageing. *J. Physiol.* 590 3305–3316. 10.1113/jphysiol.2012.229690 22570377 PMC3459044

[B81] Laurent S. Boutouyrie P. (2020). Arterial stiffness and hypertension in the elderly. *Front. Cardiovasc. Med.* 7:544302. 10.3389/fcvm.2020.544302 33330638 PMC7673379

[B82] Lee J. Shen M. Parajuli N. Oudit G. Y. Mcmurtry M. S. Kassiri Z. (2014). Gender-dependent aortic remodelling in patients with bicuspid aortic valve-associated thoracic aortic aneurysm. *J. Mol. Med.* 92 939–949. 10.1007/s00109-014-1178-6 24893666

[B83] Linton M. R. F. Yancey P. G. Davies S. S. Jerome W. G. Linton E. F. Song W. L., et al. (eds) (2000). “The role of lipids and lipoproteins in atherosclerosis,” in *Endotext*, (South Dartmouth, MA: MDText.com, Inc).

[B84] Loehr L. R. Meyer M. L. Poon A. K. Selvin E. Palta P. Tanaka H. (2016). Prediabetes and diabetes are associated with arterial stiffness in older adults: the ARIC Study. *Am. J. Hypertens.* 29 1038–1045. 27068705 10.1093/ajh/hpw036 PMC4978227

[B85] London G. M. Guérin A. P. Marchais S. J. Métivier F. Pannier B. Adda H. (2003). Arterial media calcification in end-stage renal disease: impact on all-cause and cardiovascular mortality. *Nephrol. Dial. Transplant.* 18 1731–1740. 10.1093/ndt/gfg414 12937218

[B86] London G. M. Guerin A. P. Pannier B. Marchais S. J. Stimpel M. (1995). Influence of sex on arterial hemodynamics and blood pressure. Role of body height. *Hypertension* 26 514–519. 7649591 10.1161/01.hyp.26.3.514

[B87] Luttrell M. Kim H. Shin S. Y. Holly D. Massett M. P. Woodman C. R. (2020). Heterogeneous effect of aging on vasorelaxation responses in large and small arteries. *Physiol. Rep.* 8:e14341. 10.14814/phy2.14341 PMC6971410 31960593

[B88] Ma Z. Mao C. Jia Y. Fu Y. Kong W. (2020). Extracellular matrix dynamics in vascular remodeling. *Am. J. Physiol. Cell Physiol.* 319 C481–C499. 32579472 10.1152/ajpcell.00147.2020 PMC7509265

[B89] Mackey R. H. Venkitachalam L. Sutton-Tyrrell K. (2007). Calcifications, arterial stiffness and atherosclerosis. *Adv. Cardiol.* 44 234–244. 10.1159/000096744 17075212

[B90] Mancia G. Fagard R. Narkiewicz K. Redon J. Zanchetti A. Böhm M. (2013). 2013 ESH/ESC guidelines for the management of arterial hypertension: the Task Force for the management of arterial hypertension of the European Society of Hypertension (ESH) and of the European Society of Cardiology (ESC). *Eur. Heart J.* 34 2159–2219. 23771844 10.1093/eurheartj/eht151

[B91] Maurel E. Shuttleworth C. A. Bouissou H. (1987). Interstitial collagens and ageing in human aorta. *Virchows Arch. A Pathol. Anat. Histopathol.* 410 383–390. 10.1007/bf00712757 3103320

[B92] Mceniery C. M. Mcdonnell B. J. So A. Aitken S. Bolton C. E. Munnery M. (2009). Aortic calcification is associated with aortic stiffness and isolated systolic hypertension in healthy individuals. *Hypertension* 53 524–531. 10.1161/HYPERTENSIONAHA.108.126615 19171791

[B93] Mceniery C. M. Wallace S. Mackenzie I. S. Mcdonnell B. Yasmin, Newby D. E. (2006). Endothelial function is associated with pulse pressure, pulse wave velocity, and augmentation index in healthy humans. *Hypertension* 48 602–608. 10.1161/01.hyp.0000239206.64270.5f 16940223

[B94] Medley T. L. Cole T. J. Gatzka C. D. Wang W. Y. Dart A. M. Kingwell B. A. (2002). Fibrillin-1 genotype is associated with aortic stiffness and disease severity in patients with coronary artery disease. *Circulation* 105 810–815. 10.1161/hc0702.104129 11854120

[B95] Melo E. S. F. V. Almonfrey F. B. Freitas C. M. N. Fonte F. K. Sepulvida M. B. C. Almada-Filho C. M. (2021). Association of body composition with arterial stiffness in long-lived people. *Arq. Bras. Cardiol.* 117 457–462. 10.36660/abc.20190774 34287568 PMC8462957

[B96] Millasseau S. C. Stewart A. D. Patel S. J. Redwood S. R. Chowienczyk P. J. (2005). Evaluation of carotid-femoral pulse wave velocity. *Hypertension* 45 222–226. 15642772 10.1161/01.HYP.0000154229.97341.d2

[B97] Mitchell G. F. Parise H. Benjamin E. J. Larson M. G. Keyes M. J. Vita J. A. (2004). Changes in arterial stiffness and wave reflection with advancing age in healthy men and women. *Hypertension* 43 1239–1245. 10.1161/01.HYP.0000128420.01881.aa 15123572

[B98] Moreau K. L. Hildreth K. L. (2014). Vascular aging across the menopause transition in healthy women. *Adv. Vasc. Med.* 2014:204390. 10.1155/2014/204390 PMC4433172 25984561

[B99] Moreau K. L. Meditz A. Deane K. D. Kohrt W. M. (2012). Tetrahydrobiopterin improves endothelial function and decreases arterial stiffness in estrogen-deficient postmenopausal women. *Am. J. Physiol. Heart Circ. Physiol.* 302 H1211–H1218. 10.1152/ajpheart.01065.2011 22245769 PMC3311456

[B100] Mueller N. T. Noya-Alarcon O. Contreras M. Appel L. J. Dominguez-Bello M. G. (2018). Association of age with blood pressure across the lifespan in isolated yanomami and yekwana villages. *JAMA Cardiol.* 3 1247–1249. 10.1001/jamacardio.2018.3676 30427998 PMC6583094

[B101] Nelson A. J. Worthley S. G. Cameron J. D. Willoughby S. R. Piantadosi C. Carbone A. (2009). Cardiovascular magnetic resonance-derived aortic distensibility: validation and observed regional differences in the elderly. *J. Hypertens.* 27 535–542. 19330913 10.1097/hjh.0b013e32831e4599

[B102] Nethononda R. M. Lewandowski A. J. Stewart R. Kylinterias I. Whitworth P. Francis J. (2015). Gender specific patterns of age-related decline in aortic stiffness: a cardiovascular magnetic resonance study including normal ranges. *J. Cardiovasc. Magn. Reson.* 17:20. 10.1186/s12968-015-0126-0 25827408 PMC4332729

[B103] Nosaka T. Tanaka H. Watanabe I. Sato M. Matsuda M. (2003). Influence of regular exercise on age-related changes in arterial elasticity: mechanistic insights from wall compositions in rat aorta. *Can. J. Appl. Physiol.* 28 204–212. 10.1139/h03-016 12825330

[B104] Ogola B. O. Zimmerman M. A. Clark G. L. Abshire C. M. Gentry K. M. Miller K. S. (2018). New insights into arterial stiffening: does sex matter? *Am. J. Physiol. Heart Circ. Physiol.* 315 H1073–H1087. 10.1152/ajpheart.00132.2018 30028199 PMC6415742

[B105] O’Rourke M. F. Staessen J. A. Vlachopoulos C. Duprez D. Plante G. ÉE. (2002). Clinical applications of arterial stiffness; definitions and reference values. *Am. J. Hypertens.* 15 426–444. 10.1016/s0895-7061(01)02319-6 12022246

[B106] Pagani M. Mirsky I. Baig H. Manders W. T. Kerkhof P. Vatner S. F. (1979). Effects of age on aortic pressure-diameter and elastic stiffness-stress relationships in unanesthetized sheep. *Circ. Res.* 44 420–429. 10.1161/01.res.44.3.420 104801

[B107] Pannier B. M. Avolio A. P. Hoeks A. Mancia G. Takazawa K. (2002). Methods and devices for measuring arterial compliance in humans. *Am. J. Hypertens.* 15 743–753. 10.1016/s0895-7061(02)02962-x 12160200

[B108] Pasupuleti V. R. Arigela C. S. Gan S. H. Salam S. K. N. Krishnan K. T. Rahman N. A. (2020). A review on oxidative stress, diabetic complications, and the roles of honey polyphenols. *Oxid. Med. Cell Longev.* 2020:8878172. 10.1155/2020/8878172 33299532 PMC7704201

[B109] Pescatore L. A. Gamarra L. F. Liberman M. (2019). Multifaceted mechanisms of vascular calcification in aging. *Arterioscler. Thromb. Vasc. Biol.* 39 1307–1316. 10.1161/ATVBAHA.118.311576 31144990

[B110] Pezzini A. Del Zotto E. Giossi A. Volonghi I. Costa P. Padovani A. (2012). Transforming growth factor beta signaling perturbation in the Loeys-Dietz syndrome. *Curr. Med. Chem.* 19 454–460. 22335518 10.2174/092986712803414286

[B111] Pietri P. Vlachopoulos C. Chrysohoou C. Lazaros G. Masoura K. Ioakeimidis N. (2015). Deceleration of age-related aortic stiffening in a population with high longevity rates: the IKARIA study. *J. Am. Coll. Cardiol.* 66 1842–1843. 10.1016/j.jacc.2015.07.070 26483111

[B112] Pirro M. Schillaci G. Paltriccia R. Bagaglia F. Menecali C. Mannarino M. R. (2006). Increased ratio of CD31+/CD42- microparticles to endothelial progenitors as a novel marker of atherosclerosis in hypercholesterolemia. *Arterioscler. Thromb. Vasc. Biol.* 26 2530–2535. 10.1161/01.ATV.0000243941.72375.15 16946129

[B113] Podlutsky A. J. Khritankov A. M. Ovodov N. D. Austad S. N. (2005). A new field record for bat longevity. *J. Gerontol. A Biol. Sci. Med. Sci.* 60 1366–1368. 10.1093/gerona/60.11.1366 16339320

[B114] Poznyak A. Grechko A. V. Poggio P. Myasoedova V. A. Alfieri V. Orekhov A. N. (2020). The diabetes mellitus-atherosclerosis connection: the role of lipid and glucose metabolism and chronic inflammation. *Int. J. Mol. Sci.* 21:1835. 10.3390/ijms21051835 32155866 PMC7084712

[B115] Prenner S. B. Chirinos J. A. (2015). Arterial stiffness in diabetes mellitus. *Atherosclerosis* 238 370–379. 25558032 10.1016/j.atherosclerosis.2014.12.023

[B116] Qiu H. Depre C. Ghosh K. Resuello R. G. Natividad F. F. Rossi F. (2007a). Mechanism of gender-specific differences in aortic stiffness with aging in nonhuman primates. *Circulation* 116 669–676. 10.1161/CIRCULATIONAHA.107.689208 17664374

[B117] Qiu H. Tian B. Resuello R. G. Natividad F. F. Peppas A. Shen Y. T. (2007b). Sex-specific regulation of gene expression in the aging monkey aorta. *Physiol. Genomics* 29 169–180. 10.1152/physiolgenomics.00229.2006 17456900

[B118] Qiu H. Zhu Y. Sun Z. Trzeciakowski J. P. Gansner M. Depre C. (2010). Short communication: vascular smooth muscle cell stiffness as a mechanism for increased aortic stiffness with aging. *Circ. Res.* 107 615–619. 10.1161/CIRCRESAHA.110.221846 20634486 PMC2936100

[B119] Quesada V. Freitas-Rodriguez S. Miller J. Perez-Silva J. G. Jiang Z. F. Tapia W. (2019). Giant tortoise genomes provide insights into longevity and age-related disease. *Nat. Ecol. Evol.* 3 87–95. 10.1038/s41559-018-0733-x 30510174 PMC6314442

[B120] Ravussin E. Redman L. M. Rochon J. Das S. K. Fontana L. Kraus W. E. (2015). A 2-year randomized controlled trial of human caloric restriction: feasibility and effects on predictors of health span and longevity. *J. Gerontol. A Biol. Sci. Med. Sci.* 70 1097–1104. 10.1093/gerona/glv057 26187233 PMC4841173

[B121] Reddy G. K. (2004). Cross-linking in collagen by nonenzymatic glycation increases the matrix stiffness in rabbit achilles tendon. *Exp. Diabesity Res.* 5 143–153. 15203885 10.1080/15438600490277860 PMC2496877

[B122] Redei E. E. Mehta N. S. (2015). Blood transcriptomic markers for major depression: from animal models to clinical settings. *Ann. N. Y. Acad. Sci.* 1344 37–49. 10.1111/nyas.12748 25823952

[B123] Redman L. M. Smith S. R. Burton J. H. Martin C. K. Il’yasova D. Ravussin E. (2018). Metabolic slowing and reduced oxidative damage with sustained caloric restriction support the rate of living and oxidative damage theories of aging. *Cell Metab.* 27 805.e4–815.e4. 10.1016/j.cmet.2018.02.019 29576535 PMC5886711

[B124] Rerkpattanapipat P. D’agostino R. B. Jr. Link K. M. Shahar E. Lima J. A. Bluemke D. A. (2009). Location of arterial stiffening differs in those with impaired fasting glucose versus diabetes: implications for left ventricular hypertrophy from the Multi-Ethnic Study of Atherosclerosis. *Diabetes Metab. Res. Rev.* 58 946–953. 10.2337/db08-1192 19136657 PMC2661581

[B125] Rizzoni D. Porteri E. Guelfi D. Muiesan M. L. Valentini U. Cimino A. (2001). Structural alterations in subcutaneous small arteries of normotensive and hypertensive patients with non–insulin-dependent diabetes mellitus. *Circulation* 103 1238–1244. 11238267 10.1161/01.cir.103.9.1238

[B126] Rogers I. S. Massaro J. M. Truong Q. A. Mahabadi A. A. Kriegel M. F. Fox C. S. (2013). Distribution, determinants, and normal reference values of thoracic and abdominal aortic diameters by computed tomography (from the Framingham Heart Study). *Am. J. Cardiol.* 111 1510–1516. 10.1016/j.amjcard.2013.01.306 23497775 PMC3644324

[B127] Rogers W. J. Hu Y. L. Coast D. Vido D. A. Kramer C. M. Pyeritz R. E. (2001). Age-associated changes in regional aortic pulse wave velocity. *J. Am. Coll. Cardiol.* 38 1123–1129. 10.1016/s0735-1097(01)01504-2 11583892

[B128] Safar M. E. Asmar R. Benetos A. Blacher J. Boutouyrie P. Lacolley P. (2018). Interaction between hypertension and arterial stiffness. *Hypertension* 72 796–805. 10.1161/hypertensionaha.118.11212 30354723

[B129] Sans M. Moragas A. (1993). Mathematical morphologic analysis of the aortic medial structure. Biomechanical implications. *Anal. Quant. Cytol. Histol.* 15 93–100. 8318132

[B130] Santhanam L. Tuday E. C. Webb A. K. Dowzicky P. Kim J. H. Oh Y. J. (2010). Decreased S-nitrosylation of tissue transglutaminase contributes to age-related increases in vascular stiffness. *Circ. Res.* 107 117–125. 10.1161/CIRCRESAHA.109.215228 20489165

[B131] Santos-Parker J. R. Larocca T. J. Seals D. R. (2014). Aerobic exercise and other healthy lifestyle factors that influence vascular aging. *Adv. Physiol. Educ.* 38 296–307. 25434012 10.1152/advan.00088.2014 PMC4315444

[B132] Schillaci G. Verdecchia P. Borgioni C. Ciucci A. Porcellati C. (1998). Early cardiac changes after menopause. *Hypertension* 32 764–769. 9774377 10.1161/01.hyp.32.4.764

[B133] Schlatmann T. J. M. Becker A. E. (1977). Histologic changes in the normal aging aorta: implications for dissecting aortic aneurysm. *Am. J. Cardiol.* 39 13–20. 831420 10.1016/s0002-9149(77)80004-0

[B134] Schleicher E. D. Wagner E. Nerlich A. G. (1997). Increased accumulation of the glycoxidation product N(epsilon)-(carboxymethyl)lysine in human tissues in diabetes and aging. *J. Clin. Invest.* 99 457–468. 9022079 10.1172/JCI119180 PMC507819

[B135] Schofield I. Malik R. Izzard A. Austin C. Heagerty A. (2002). Vascular structural and functional changes in type 2 diabetes mellitus. *Circulation* 106 3037–3043. 12473548 10.1161/01.cir.0000041432.80615.a5

[B136] Schram M. T. Henry R. M. van Dijk R. A. Kostense P. J. Dekker J. M. Nijpels G. (2004). Increased central artery stiffness in impaired glucose metabolism and type 2 diabetes. *Hypertension* 43 176–181. 10.1161/01.hyp.0000111829.46090.92 14698999

[B137] Sehgel N. L. Sun Z. Hong Z. Hunter W. C. Hill M. A. Vatner D. E. (2015a). Augmented vascular smooth muscle cell stiffness and adhesion when hypertension is superimposed on aging. *Hypertension* 65 370–377. 10.1161/HYPERTENSIONAHA.114.04456 25452471 PMC4289111

[B138] Sehgel N. L. Vatner S. F. Meininger G. A. (2015b). “Smooth muscle cell stiffness syndrome”-revisiting the structural basis of arterial stiffness. *Front. Physiol.* 6:335. 10.3389/fphys.2015.00335 26635621 PMC4649054

[B139] Sehgel N. L. Zhu Y. Sun Z. Trzeciakowski J. P. Hong Z. Hunter W. C. (2013). Increased vascular smooth muscle cell stiffness: a novel mechanism for aortic stiffness in hypertension. *Am. J. Physiol. Heart Circ. Physiol.* 305 H1281–H1287. 23709594 10.1152/ajpheart.00232.2013 PMC3840243

[B140] Shadwick R. E. Gosline J. M. (1994). Arterial mechanics in the fin whale suggest a unique hemodynamic design. *Am. J. Physiol.* 267 R805–R818. 10.1152/ajpregu.1994.267.3.R805 8092327

[B141] Siasos G. Chrysohoou C. Tousoulis D. Oikonomou E. Panagiotakos D. Zaromitidou M. (2013). The impact of physical activity on endothelial function in middle-aged and elderly subjects: the Ikaria study. *Hellenic J. Cardiol.* 54 94–101. 23557608

[B142] Sloop G. D. Weidman J. J. Shecterle L. M. (2015). The interplay of aging, aortic stiffness, and blood viscosity in atherogenesis. *J. Cardiol. Ther.* 2 350–354.

[B143] Smulyan H. Asmar R. G. Rudnicki A. London G. M. Safar M. E. (2001). Comparative effects of aging in men and women on the properties of the arterial tree. *J. Am. Coll. Cardiol.* 37 1374–1380. 10.1016/s0735-1097(01)01166-4 11300449

[B144] Sokolis D. P. (2007). Passive mechanical properties and structure of the aorta: segmental analysis. *Acta Physiol.* 190 277–289. 10.1111/j.1748-1716.2006.01661.x 17635348

[B145] Sorescu G. P. Song H. Tressel S. L. Hwang J. Dikalov S. Smith D. A. (2004). Bone morphogenic protein 4 produced in endothelial cells by oscillatory shear stress induces monocyte adhesion by stimulating reactive oxygen species production from a nox1-based NADPH oxidase. *Circ. Res.* 95 773–779. 10.1161/01.RES.0000145728.22878.45 15388638

[B146] Staessen J. A. Van Der Heijden-Spek J. J. Safar M. E. Den Hond E. Gasowski J. Fagard R. H. (2001). Menopause and the characteristics of the large arteries in a population study. *J. Hum. Hypertens.* 15 511–518. 11494087 10.1038/sj.jhh.1001226

[B147] Stehouwer C. D. A. Henry R. M. A. Ferreira I. (2008). Arterial stiffness in diabetes and the metabolic syndrome: a pathway to cardiovascular disease. *Diabetologia* 51:527. 10.1007/s00125-007-0918-3 18239908

[B148] Sugawara J. Tomoto T. Lin H.-F. Chen C.-H. Tanaka H. (2018). Aortic reservoir function of Japanese female pearl divers. *J. Appl. Physiol.* 125 1901–1905. 10.1152/japplphysiol.00466.2018 30070611

[B149] Sun Z. (2015). Aging, arterial stiffness, and hypertension. *Hypertension* 65 252–256. 25368028 10.1161/HYPERTENSIONAHA.114.03617 PMC4288978

[B150] Taviani V. Hickson S. S. Hardy C. J. Mceniery C. M. Patterson A. J. Gillard J. H. (2011). Age-related changes of regional pulse wave velocity in the descending aorta using Fourier velocity encoded M-mode. *Magn. Reson. Med.* 65 261–268. 10.1002/mrm.22590 20878761

[B151] Trache A. Massett M. P. Woodman C. R. (2020). “Chapter Six - Vascular smooth muscle stiffness and its role in aging,” in *Current Topics in Membranes*, eds Levitan I. Trache A. (Cambridge, MA: Academic Press), 217–253. 10.1016/bs.ctm.2020.08.008 33837694

[B152] Trepanowski J. F. Canale R. E. Marshall K. E. Kabir M. M. Bloomer R. J. (2011). Impact of caloric and dietary restriction regimens on markers of health and longevity in humans and animals: a summary of available findings. *Nutr. J.* 10:107. 10.1186/1475-2891-10-107 21981968 PMC3200169

[B153] Tsimploulis A. Sheriff H. M. Lam P. H. Dooley D. J. Anker M. S. Papademetriou V. (2017). Systolic-diastolic hypertension versus isolated systolic hypertension and incident heart failure in older adults: insights from the Cardiovascular Health Study. *Int. J. Cardiol.* 235 11–16. 28291625 10.1016/j.ijcard.2017.02.139 PMC6454896

[B154] Urbina E. M. Wadwa R. P. Davis C. Snively B. M. Dolan L. M. Daniels S. R. (2010). Prevalence of increased arterial stiffness in children with type 1 diabetes mellitus differs by measurement site and sex: the SEARCH for diabetes in youth Study. *J. Pediatr.* 156 731.e1–737.e1. 10.1016/j.jpeds.2009.11.011 20097360

[B155] Van Bussel F. C. Van Bussel B. C. Hoeks A. P. Op ’T Roodt J. Henry R. M. Ferreira I. (2015). A control systems approach to quantify wall shear stress normalization by flow-mediated dilation in the brachial artery. *PLoS One* 10:e0115977. 10.1371/journal.pone.0115977 25693114 PMC4333124

[B156] Van Popele N. M. Grobbee D. E. Bots M. L. Asmar R. Topouchian J. Reneman R. S. (2001). Association between arterial stiffness and atherosclerosis: the Rotterdam Study. *Stroke* 32 454–460. 10.1161/01.str.32.2.454 11157182

[B157] Vatner S. F. Zhang J. Oydanich M. Berkman T. Naftalovich R. Vatner D. E. (2020). Healthful aging mediated by inhibition of oxidative stress. *Ageing Res. Rev.* 64:101194. 10.1016/j.arr.2020.101194 33091597 PMC7710569

[B158] Verwoert G. C. Franco O. H. Hoeks A. P. Reneman R. S. Hofman A. Cm V. D. (2014). Arterial stiffness and hypertension in a large population of untreated individuals: the Rotterdam Study. *J. Hypertens.* 32 1606–1612. 10.1097/HJH.0000000000000237 24886821

[B159] Wagenseil J. E. Mecham R. P. (2012). Elastin in large artery stiffness and hypertension. *J. Cardiovasc. Transl. Res.* 5 264–273. 10.1007/s12265-012-9349-8 22290157 PMC3383658

[B160] Westenberg J. J. Scholte A. J. Vaskova Z. Van Der Geest R. J. Groenink M. Labadie G. (2011). Age-related and regional changes of aortic stiffness in the Marfan syndrome: assessment with velocity-encoded MRI. *J. Magn. Reson. Imaging* 34 526–531. 10.1002/jmri.22646 21761466

[B161] Wheeler J. B. Mukherjee R. Stroud R. E. Jones J. A. Ikonomidis J. S. (2015). Relation of murine thoracic aortic structural and cellular changes with aging to passive and active mechanical properties. *J. Am. Heart Assoc.* 4:e001744. 10.1161/JAHA.114.001744 25716945 PMC4392448

[B162] Wilkinson I. B. Qasem A. Mceniery C. M. Webb D. J. Avolio A. P. Cockcroft J. R. (2002). Nitric oxide regulates local arterial distensibility in vivo. *Circulation* 105 213–217. 10.1161/hc0202.101970 11790703

[B163] Wilson D. P. (2000). “Is atherosclerosis a pediatric disease?,” in *Endotext*, eds Feingold K. R. Anawalt B. Boyce A. Chrousos G. De Herder W. W. Dhatariya K. (South Dartmouth, MA: MDText.com, Inc).

[B164] Wolinsky H. (1970). Response of the rat aortic media to hypertension. Morphological and chemical studies. *Circ. Res.* 26 507–522. 10.1161/01.res.26.4.507 5435712

[B165] Woodward M. (2019). Cardiovascular disease and the female disadvantage. *Int. J. Environ. Res. Public Health* 16:1165. 10.3390/ijerph16071165 PMC6479531 30939754

[B166] Yan L. Gao S. Ho D. Park M. Ge H. Wang C. (2013). Calorie restriction can reverse, as well as prevent, aging cardiomyopathy. *Age* 35 2177–2182. 10.1007/s11357-012-9508-5 23334601 PMC3825004

[B167] Yan L. Park J. Y. Dillinger J. G. De Lorenzo M. S. Yuan C. Lai L. (2012). Common mechanisms for calorie restriction and adenylyl cyclase type 5 knockout models of longevity. *Aging Cell* 11 1110–1120. 10.1111/acel.12013 23020244 PMC3646327

[B168] Yasmin, Wallace S. Mceniery C. M. Dakham Z. Pusalkar P. Maki-Petaja K. (2005). Matrix metalloproteinase-9 (MMP-9), MMP-2, and serum elastase activity are associated with systolic hypertension and arterial stiffness. *Arterioscler. Thromb. Vasc. Biol.* 25 372–378. 10.1161/01.ATV.0000151373.33830.41 15556929

[B169] Yoon B. K. Oh W. J. Kessel B. Roh C. R. Choi D. Lee J. H. (2001). 17Beta-estradiol inhibits proliferation of cultured vascular smooth muscle cells induced by lysophosphatidylcholine via a nongenomic antioxidant mechanism. *Menopause* 8 58–64. 10.1097/00042192-200101000-00010 11201517

[B170] Yu S. Mceniery C. M. (2020). Central versus peripheral artery stiffening and cardiovascular risk. *Arterioscler. Thromb. Vasc. Biol.* 40 1028–1033. 10.1161/atvbaha.120.313128 32188277

[B171] Zagura M. Kals J. Serg M. Kampus P. Zilmer M. Jakobson M. (2012). Structural and biochemical characteristics of arterial stiffness in patients with atherosclerosis and in healthy subjects. *Hypertens. Res.* 35 1032–1037. 10.1038/hr.2012.88 22739422

[B172] Zhang J. Zhao X. Vatner D. E. Mcnulty T. Bishop S. Sun Z. (2016). Extracellular matrix disarray as a mechanism for greater abdominal versus thoracic aortic stiffness with aging in primates. *Arterioscler. Thromb. Vasc. Biol.* 36 700–706. 10.1161/atvbaha.115.306563 26891739 PMC4808390

[B173] Zheng M. Zhang X. Chen S. Song Y. Zhao Q. Gao X. (2020). Arterial stiffness preceding diabetes. *Circ. Res.* 127 1491–1498. 10.1161/circresaha.120.317950 32985370

